# Rearming mesenchymal stem cells with engineering strategies to combat cancer

**DOI:** 10.3389/fimmu.2026.1845224

**Published:** 2026-06-08

**Authors:** Ting Yu, Sikan Jin, Rui Xu, Yaqi Zhang, Xianyao Wang

**Affiliations:** Department of Immunology & Key Laboratory of Cancer Prevention and Treatment of Guizhou Province, Zunyi Medical University, Zunyi, China

**Keywords:** antitumor, genetic engineering, mesenchymal stem cells, target therapy, tumor micoenvironment, tumor tropism

## Abstract

Mesenchymal stromal/stem cells (MSCs) are garnering increasing attention as promising tumor-targeted delivery vehicles owing to their ability to sense inflammatory signals and access tumor-adjacent stromal niches. However, their plasticity in the tumor microenvironment also enables pro-tumor programs that promote angiogenesis, matrix remodeling, immune suppression, stemness, and therapy resistance. This review summarizes the molecular “homing hierarchy” that tumors exploit to recruit MSCs, primarily through the CXCL12/CXCR4 signaling axis, and integrates key pathways implicated in MSC-driven tumor progression. The focus is on “turning enemies into allies”: engineering MSCs into programmable intratumoral factories and carriers to deliver defined anticancer payloads, including suicide gene systems, pro-apoptotic and anti-angiogenic effectors, immune-stimulatory cytokines, localized checkpoint blockade formats, and oncolytic viruses that combine intratumoral amplification with immunogenic remodeling. Furthermore, we summarize antigen-directed strategies aimed at enhancing delivery efficacy and strategies to precisely control the release of antitumor components to minimize off-target effects. Additionally, we discuss the formidable challenges associated with harnessing MSCs for antitumor therapy. Looking ahead, further optimization of MSC-based antitumor strategies through advanced genetic engineering and combination therapies holds the potential to significantly enhance their clinical efficacy.

## 1 Introduction

Mesenchymal stem/stromal cells (MSCs) are multipotent cells of mesodermal origin, characterized by self-renewal capacity, multilineage differentiation potential, and immunomodulatory and tissue-homing properties (1). They can differentiate into adipocytes, osteoblasts, and chondrocytes and exert therapeutic effects primarily through paracrine signaling and extracellular vesicle (EV)-mediated mechanisms (2).

Tumor development occurs within a dynamic tumor microenvironment (TME) composed of malignant cells, immune cells, cancer-associated fibroblasts, endothelial cells, and MSCs embedded in an extracellular matrix and soluble signaling milieu (3). These components interact to regulate tumor proliferation, invasion, metastasis, and therapeutic responses. The TME is characterized by marked heterogeneity, immunosuppression, abnormal vascularization, hypoxia, and metabolic reprogramming, collectively promoting tumor progression and resistance to therapy (4, 5).

Within this intricate microenvironment, MSCs have emerged as enigmatic players that exert complex and often contradictory effects on tumor progression. Evidence suggests a tumor-promoting role for MSCs, which enhance tumor cell proliferation, invasion, metastasis, and therapy resistance through mechanisms ranging from cell fusion to paracrine signaling and EV-mediated communication (6, 7). For instance, fusion with malignant cells may endow the latter with enhanced stem cell-like properties and metastatic capacities (8). Concurrently, bioactive molecules secreted by MSCs modulate immune cell function and promote angiogenesis, thereby supporting tumor growth. Conversely, some studies have suggested that MSCs may suppress malignant behavior after fusion with specific tumor cells or exert antitumor effects through immunomodulation and apoptosis induction (9). Furthermore, by leveraging the tumor tropism and paracrine capabilities of MSCs, researchers are exploring their engineering as carriers for the targeted delivery of anticancer drugs or regulatory factors, offering novel therapeutic strategies for cancer treatment (10).

Thus, MSCs can assume opposing roles within the TME, acting as either tumor-promoting “accomplices” or tumor-suppressing “killers,” depending on a constellation of factors, including their tissue of origin, specific tumor type, microenvironmental signals, and timing of their intervention. This functional duality not only reveals the tension between their beneficial roles in tissue repair and their potential dark side in malignancy but also highlights the critical need to fully elucidate the underlying mechanisms. In this review, we systematically examine the double-edged role of MSCs in tumor immunity, focusing on the mechanisms through which they promote tumor growth and metastasis as well as their potential to suppress tumor progression. We also discuss emerging strategies for engineering MSCs to shift the balance toward anticancer therapy, transforming these versatile cells from unintended accomplices into purposeful anticancer agents.

## 2 TME recruitment of MSCs

### 2.1 Sources of cytokines

Within the TME, cytokines function as essential signaling molecules that establish a complex and dynamic intercellular communication network (11). This network is not established by a single cell type; rather, it reflects the collective contributions of all major TME constituents, including tumor cells, immune cells, CAFs, and vascular endothelial cells.

**Figure 1:**
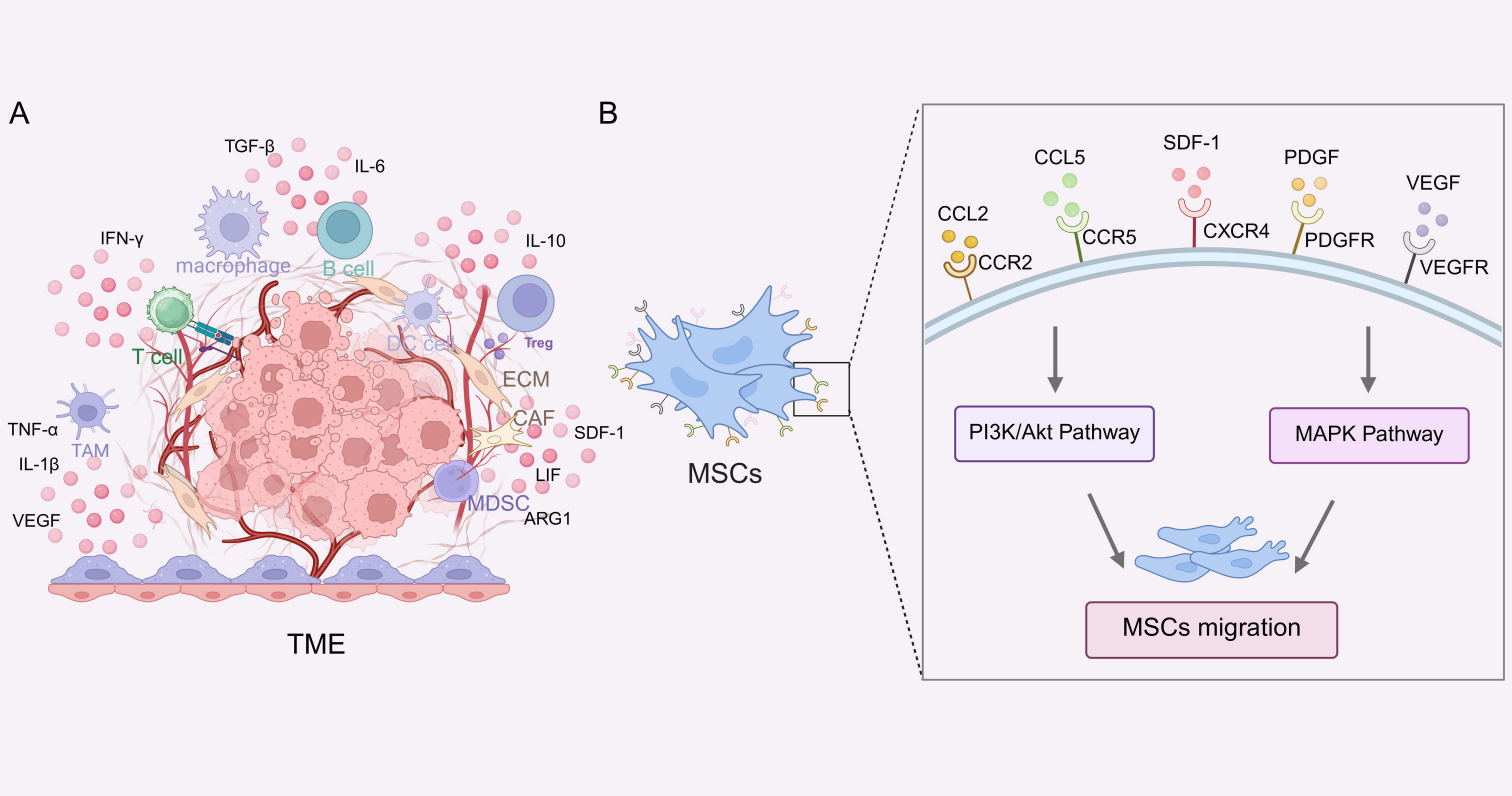
Intricate cytokine network within the tumor microenvironment (TME) and the underlying mechanisms regulating mesenchymal stem cell (MSC) tumor tropism **(A)** The TME comprises tumor cells, immune cells (e.g. T cells, B cells, dendritic cells, tumor-associated macrophages [TAMs], regulatory T cells [Tregs], and myeloid-derived suppressor cells [MDSCs]), and stromal components, such as cancer-associated fibroblasts (CAFs) and the extracellular matrix (ECM). These cellular components collectively secrete a broad range of cytokines and mediators, including interleukin-6 (IL-6), IL-10, transforming growth factor-beta (TGF-β), tumor necrosis factor-alpha (TNF-α), interleukin-1 beta (IL-1β), vascular endothelial growth factor (VEGF), stromal cell-derived factor 1 (c-x-c motif chemokine ligand 12, SDF-1/CXCL12), leukemia inhibitory factor (LIF), and arginase 1 (ARG1), forming a complex signaling network that promotes immunosuppression, angiogenesis, and tumor progression. **(B)** MSC tumor tropism is mediated by multiple chemokines and growth factor signaling axes. Chemokines such as CXCL12, CCL2, and CCL5 interact with their respective receptors (CXCR4, CCR2, and CCR5), while growth factors including platelet-derived growth factor (PDGF) and vascular endothelial growth factor (VEGF) signal through platelet-derived growth factor receptor (PDGFR) and vascular endothelial growth factor receptor (VEGFR). These upstream cues activate intracellular pathways, particularly phosphoinositide 3-kinase/protein kinase b (PI3K/Akt) and mitogen-activated protein kinase (MAPK) signaling, thereby promoting cytoskeletal remodeling and directional migration of MSCs toward the tumor site.

Tumor cells are a prominent source of pro-tumorigenic cytokines. They secrete factors such as interleukin-6 (IL-6), IL-8, vascular endothelial growth factor (VEGF), and transforming growth factor-beta (TGF-β), which promote proliferation, survival, and epithelial-mesenchymal transition and suppress local immune surveillance (12). Immune cells are the primary source of both inflammatory and immunoregulatory cytokines. Tumor-associated macrophages (TAMs) abundantly produce IL-1β, IL-6, IL-10, TNF-α, and VEGF, which directly stimulate tumor growth and amplify immunosuppression and angiogenesis (13). Tregs further dampen antitumor immunity by releasing inhibitory cytokines, including IL-10 and TGF-β, whereas myeloid-derived suppressor cells (MDSCs) contribute additional immunosuppressive mediators, such as IL-10, TGF-β, and ARG1, effectively suppressing T-cell and natural killer (NK) cell activity (14). Stromal cells, particularly CAFs, are essential components of the cytokine network. Once activated, CAFs secrete a broad spectrum of factors, such as CXCL12 (SDF-1), IL-6, IL-8, LIF, and VEGF (3) (**Figure 1A**).

Together, these cytokine networks coordinate immune suppression, angiogenesis and tumor progression. The CXCL12/CXCR4 chemokine system directs immune cell trafficking and serves as the primary chemotactic cue for recruiting stromal precursor cells, including MSCs, into the TME.

### 2.2 Mechanisms of MSC tumor tropism

The directed migration of MSCs toward tumor tissue, termed tumor tropism, represents a critical initiating event that drives their involvement in tumor progression. Although the CXCL12/CXCR4 axis has long been recognized as the dominant chemotactic pathway, accumulating evidence indicates that multiple signaling systems contribute to MSC recruitment. Inflammatory chemokines, including CCL2 and CCL5, act through their respective receptors, CCR2 and CCR5, and operate in parallel with the CXCL12 axis (15). These pathways are frequently coupled with myeloid cell recruitment and the establishment of an immunosuppressive microenvironment, thereby creating a multichannel navigation system that guides MSC migration. Additionally, tumors and their associated stroma release growth factors such as PDGF and VEGF (**Figure 1B**). These molecules, traditionally known for their roles in angiogenesis, also function as migration-related signals and modulators of endothelial barrier function. They alter vascular permeability and adhesion properties, thereby influencing the accessibility of the endothelial lining for cell extravasation and subsequent tissue infiltration (16). More critically, hypoxia, which is prevalent in tumor cores, stabilizes hypoxia-inducible factor 1 alpha (HIF-1α), upregulates multiple response genes, including VEGF, and amplifies chemotactic signals, such as CXCL12 (17). This oxygen-sensitive mechanism transforms what might otherwise be localized and transient signals into a sustainably maintained recruitment program (15).

The migratory capacity of MSCs depends on a flexible repertoire of receptors and effector modules, which together determine their ability to integrate multifactorial signals from the TME. CXCR4 serves as a core component of this receptor apparatus, conferring sensitivity to CXCL12 and strongly correlating with tropism toward chemotactic tumor niches. Additional receptors, including CCR2, CCR5, VEGFR, and PDGFR, contribute to this integrated response, and their expression profiles vary considerably depending on the MSC tissue source and the state of inflammatory exposure. Upon receptor engagement, MSCs activate migration-associated signaling pathways, particularly PI3K/Akt and MAPK, to drive actin cytoskeleton reorganization and establish cell polarity (18). Enhanced integrin activation concurrently stabilizes adhesion to the endothelium and extracellular matrix, whereas upregulation or release of matrix-remodeling enzymes, such as matrix metalloproteinases, reduces the physical barriers imposed by the ECM. The coordinated action of these events enables transendothelial migration and subsequent advancement through the interstitial spaces. However, these cellular mechanisms operate within a dynamically evolving TME, in which multiple extrinsic factors critically influence efficacy. Vascular abnormalities and stromal stiffening, which are hallmarks of many solid tumors, substantially alter the structural pathways available for cell migration (19). Concurrently, tumor-associated fibroblasts and specialized vascular niches contribute to the spatial organization of chemotactic cues, further refining the directional guidance of infiltrating cells.

Although the CXCL12/CXCR4 axis plays a central role in the tumor tropism of MSCs, it does not predominate under all conditions. Its function is frequently shaped by synergistic, compensatory, or hierarchical interactions with other signaling pathways. In terms of signal characteristics, the CXCL12/CXCR4 axis provides a sustained and stable chemotactic gradient, which is particularly suited to the hypoxic tumor core. In contrast, inflammatory chemokine axes, such as CCL2/CCL5, respond to acute inflammation and exhibit high signal dynamics but with relatively weak persistence. These two types of signals often display spatiotemporal complementarity, with CCL2/CCL5-mediated axes guiding early recruitment, whereas the CXCL12/CXCR4 axis becomes dominant during tumor progression (20). Growth factor pathways, including those mediated by PDGF and VEGF, do not directly substitute chemokine axes. Instead, they modulate endothelial permeability and matrix remodeling, thereby indirectly influencing the efficiency of transendothelial migration and serving regulatory roles. Furthermore, the receptor expression profiles of MSCs vary depending on their tissue origin. Bone marrow-derived MSCs highly express CXCR4, whereas adipose- or umbilical cord-derived MSCs may rely more on CCR2 or PDGFR, resulting in markedly different outcomes upon single-pathway blockade. Crosstalk among these pathways further complicates the regulatory network; hypoxia-induced HIF-1α simultaneously upregulates CXCL12, VEGF, and PDGF, forming a cascade in which multiple pathways are activated concurrently, with CXCR4 serving as an integrative hub for these signals. Consequently, targeting CXCR4 alone may be insufficient to completely block MSC migration, particularly when compensatory upregulation is observed.

In summary, the recruitment of MSCs to the TME illustrates how malignancies exploit endogenous repair pathways to achieve a pathological advantage. This process integrates multiple chemotactic signals, including chemokines, growth factors, and hypoxia-driven amplifiers, which are recognized by complementary receptor arrays on MSCs and transduced for cytoskeletal reorganization and directed migration. Elucidating this molecular recruitment program revealed the fundamental mechanism of tumor-stromal crosstalk and identified potential intervention points. Targeting these components, whether at the level of chemokine receptors, signaling kinases, or downstream effectors, may provide novel strategies for disrupting the pro-tumorigenic support network sustained by infiltrating MSCs.

## 3 Determinants of MSC migration efficiency

Although MSC recruitment to tumors via chemotactic pathways, such as CXCL12/CXCR4, has been well established, migration efficiency and functional outcomes are highly context-dependent. Factors such as the MSC source, phenotypic state, tumor type, vascular accessibility, and delivery strategy collectively determine homing efficiency and subsequent functional polarization.

### 3.1 Intrinsic determinants of migration heterogeneity

Among these determinants, the intrinsic biological properties of MSCs constitute a major source of heterogeneity in their homing behavior. MSCs derived from different tissue sources (e.g., bone marrow, adipose tissue, and umbilical cord) exhibit distinct receptor expression profiles, secretory patterns, and migratory capacities (21). For example, bone marrow-derived MSCs are often considered responsive to CXCL12-rich environments; however, surface CXCR4 expression is frequently limited to only a subset of cells and may decline during ex vivo expansion, thereby weakening their chemotactic responsiveness and generating substantial batch-to-batch variations (22). In contrast, umbilical cord-derived MSCs have been reported in comparative studies to display a more robust migratory phenotype, including stronger Transwell migration in response to activated immune or inflammatory signals, partly associated with PDGF-, IGF-1-, and MMP-related pathways (23). These results suggest that MSC properties are further influenced by culture conditions, passage number, and *in vitro* stress, highlighting that migratory competence is dynamic rather than fixed.

### 3.2 Extrinsic and intervention-related modulators

In addition to intrinsic cellular properties, migration efficiency is modulated by extrinsic factors in the TME and intervention protocols. The chemotactic landscape and vascular-stromal architecture of different tumors vary considerably according to histological type, anatomical site, and disease stage; thus, the same MSC preparation may behave differently across tumor models (10). For instance, tumors with prominent stromal desmoplasia, aberrant vasculature, or elevated interstitial pressure may restrict MSC extravasation and intratumoral dispersion, even in the presence of relevant chemokines (24). Likewise, the administration route and dose critically influence biodistribution, circulation kinetics, and first-pass sequestration in filter organs, such as the lungs, liver, and spleen, thereby determining the fraction of infused cells that ultimately reach the tumor target.

### 3.3 Strategies to enhance MSC migration

The multifactorial nature of MSC tumor tropism provides a rational framework for developing strategies to enhance migration efficiency. As discussed in the preceding sections, this process is shaped by the complex interplay between intrinsic cell properties, tumor-specific microenvironmental features, and intervention-related variables. Various approaches have been developed to enhance MSC migratory capacity, addressing the limitations of heterogeneous and often suboptimal homing.

A widely explored strategy involves preconditioning, in which MSCs are exposed to specific environmental cues prior to administration to prime their migratory machinery. Hypoxic preconditioning, for instance, stabilizes HIF-1α and upregulates downstream effectors, such as CXCR4 and VEGF, thereby enhancing chemotactic sensitivity and survival upon engraftment (25). Similarly, inflammatory priming with cytokines, including TNF-α, IFN-γ, or IL-1β, has been shown to potentiate MSC responsiveness by upregulating adhesion molecules, chemokine receptors, and matrix-remodeling enzymes, thereby improving their ability to navigate inflammatory or tumor-associated signals (26). As an alternative but complementary approach, genetic engineering enables the stable modification of key homing-related molecules (27). CXCR4 overexpression has been the most extensively investigated strategy, directly enhancing MSC sensitivity to the CXCL12-rich TME (28). Additionally, engineering strategies have targeted other chemokine receptors, pro-migratory signaling mediators, or surface molecules involved in endothelial adhesion and extravasation, thereby reinforcing multiple migration cascade nodes (29).

A comprehensive understanding of MSC tumor tropism determinants is crucial for interpreting research and designing strategies to harness or counteract MSC migration for therapy. Refining preconditioning and genetic engineering, guided by mechanistic insights into MSC biology and tumor–stroma crosstalk, could improve MSC-based therapies.

## 4 The dark side: the tumor-promoting mechanism of MSCs

Although MSC recruitment to tumors is recognized as a dynamic process orchestrated by chemokines, inflammatory mediators, adhesion molecules, and vascular permissiveness, migration does not constitute a biological endpoint of MSC involvement in cancer. When MSCs traverse the vascular barrier and enter the tumor stroma, they are exposed to a highly complex niche composed of malignant cells, infiltrating immune populations, CAFs, ECM components, and persistent hypoxic and inflammatory stress. In this environment, the broad immunoregulatory capacity and pronounced phenotypic plasticity of MSCs are continuously reshaped by the local signals. Consequently, the biological outcome of MSC engraftment is not predetermined; rather, whether MSCs ultimately suppress or support tumor progression depends on their functional reprogramming after arrival. This post-migration re-education serves as a conceptual foundation for understanding the dual potential of MSCs in cancer biology.

### 4.1 Enhancing tumor cell stemness and drug resistance

**Figure 2:**
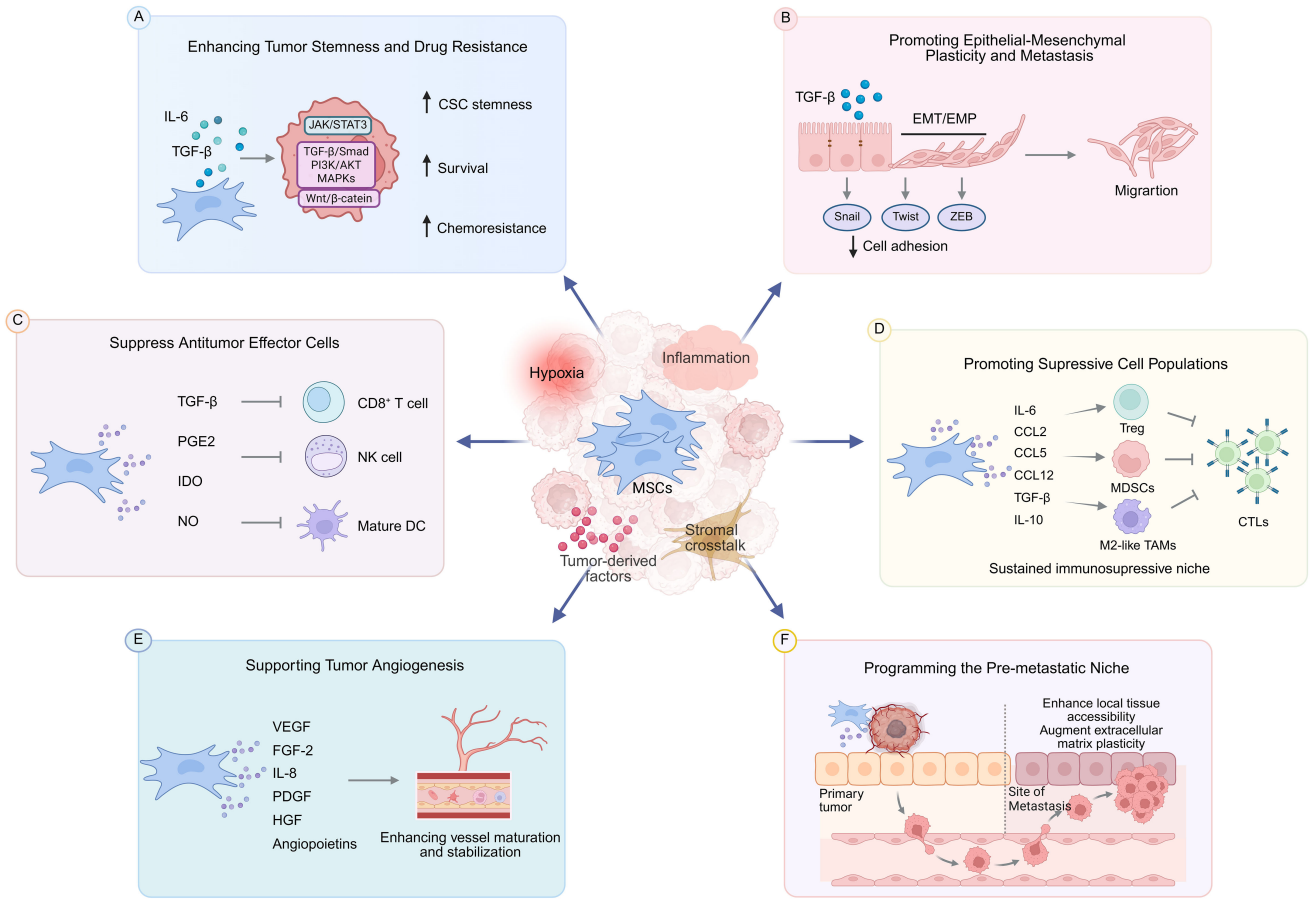
Mesenchymal stem cells (MSCs) promote tumor progression via multiple mechanisms. MSCs within the tumor microenvironment are subjected to multiple stimuli, including inflammation, hypoxia, and tumor-derived factors. These signals trigger stromal crosstalk between MSCs and other cellular components, collectively promoting tumor progression. **(A)** MSC-derived interleukin-6 (IL-6) and transforming growth factor-beta (TGF-β) activate the janus kinase/signal transducer and activator of transcription 3 (JAK/STAT3), phosphoinositide 3-kinase/protein kinase b (PI3K/AKT), mitogen-activated protein kinase (MAPK), TGF-β/Smad, and Wnt/β-catenin signaling pathways, promoting cancer stem cell (CSC) stemness, tumor cell survival, and chemoresistance. **(B)** MSC-derived TGF-β induces epithelial–mesenchymal transition and epithelial–mesenchymal plasticity (EMT/EMP) via transcription factors such as Snail, Twist, and ZEB, resulting in decreased cell adhesion and increased tumor cell migration. **(C)** MSCs suppress antitumor effector cells through TGF-β, PGE2, IDO, and NO, thereby inhibiting the maturation of CD8^+^ T cells, natural killer (NK) cells, and dendritic cells. **(D)** MSCs promote the accumulation of immunosuppressive cell populations by secreting IL-6, c-c motif chemokine ligand 2 (CCL2), CCL5, CXCL12, TGF-β, and IL-10, leading to the enrichment of Tregs, myeloid-derived suppressor cells (MDSCs), and M2-like tumor-associated macrophages (TAMs), and suppression of cytotoxic T lymphocytes (CTLs). The coordinated action of these events generates a sustained immunosuppressive microenvironment that supports tumor progression. **(E)** MSCs support tumor angiogenesis by secreting vascular endothelial growth factor (VEGF), fibroblast growth factor 2 (FGF-2), interleukin-8 (IL-8), platelet-derived growth factor (PDGF), hepatocyte growth factor (HGF), and angiopoietins, thereby promoting vascular formation, maturation, and stabilization. **(F)** MSCs contribute to pre-metastatic niche formation by increasing tissue accessibility and extracellular matrix plasticity at distant sites, thereby facilitating tumor cell colonization.

Within the TME, MSCs profoundly influence malignant progression by releasing soluble mediators that remodel intrinsic tumor cell programs. Among these, IL-6 and TGF-β function as critical signaling nodes that converge on downstream pathways to establish and sustain cancer stem cell (CSC) phenotypes and drive therapeutic resistance (30) (**Figure 2A**). These factors engage in extensive crosstalk, forming an integrated network through which stromal cues are translated into durable changes in tumor cell identity and treatment responsiveness.

Although MSCs are not the predominant source of IL-6 under basal conditions, their secretory profile is highly plastic and can be substantially reshaped by inflammatory cytokines, hypoxia, and reciprocal interactions with tumor cells. Under these conditions, MSCs become an important stromal source of IL-6, thereby reinforcing a cytokine milieu that favors tumor progression. This process is sustained by both autocrine and paracrine amplification circuits; IL-6 enhances its own production in MSCs, whereas inflammatory inputs from neighboring cells further potentiate MSC activation (7). Mechanistically, MSC-derived IL-6 activates the JAK/STAT3 pathway, promoting tumor cell proliferation, survival, and stemness, while enhancing resistance to apoptosis (31). These effects are strengthened by crosstalk between the PI3K/AKT and MAPK pathways, allowing IL-6/STAT3 signaling to function as a central hub that integrates proliferative, anti-apoptotic, and invasive programs. Evidence from multiple tumor models supports this mechanism; in gliomas, glioma-resident MSCs enhance glioma stem cell proliferation via the IL-6/gp130/STAT3 axis, and their abundance correlates with poor clinical outcomes (32).

Similar to IL-6, TGF-β secreted by MSCs serves as a pivotal mediator of tumor stemness and therapeutic resistance. One of the most important functions of TGF-β is to drive MSC differentiation into CAFs, thereby establishing a self-sustaining stromal program that actively supports malignant progression (9). In gastric cancer, approximately 20% of CAFs originate from bone marrow-derived MSCs, a process partially dependent on TGF-β and SDF-1 signaling. Similar observations have been made in breast and ovarian cancers, where MSCs acquire CAF-like phenotypes in response to TGF-β family members (33, 34). This transition is not merely a phenotypic conversion; rather, it initiates a self-sustaining circuit in which MSC-derived CAFs continue to secrete TGF-β, maintaining autocrine activation within the stroma while acting on adjacent tumor cells in a paracrine manner. At the signaling level, TGF-β initiates both canonical (SMAD2/3–SMAD4) and non-canonical (PI3K/AKT, MAPK) pathways, directly contributing to the acquisition and maintenance of stem-like phenotypes (35). In gastric cancer models, co-culture with CAFs significantly enhanced spheroid formation and stemness marker expression, an effect that was reversible by TGF-β inhibition. Beyond transcriptional regulation, TGF-β reshapes the physical properties of the TME through the Snail1/RhoA/α-smooth muscle actin axis, promoting ECM remodeling, increasing matrix stiffness, and inducing anisotropic fiber organization (36). This structural context, characterized by increased matrix stiffness, enhances intracellular tension and integrin clustering, creating a biomechanical environment conducive to the maintenance of stemness and invasive growth. The Wnt/β-catenin pathway acts as a central downstream mediator, integrating stromal signals to promote epithelial–mesenchymal transition (EMT), stemness, and therapeutic resistance (37).

In addition to their direct effects on tumor cell intrinsic programs, MSC-derived signals contribute to the establishment of an immunosuppressive and treatment-refractory microenvironment. Sustained IL-6/STAT3 signaling has been implicated in PD-L1 upregulation, recruitment of myeloid-derived suppressor cells, and polarization of macrophages toward M2-like phenotypes, thereby weakening antitumor immunity (38). In gastric cancer, cancer-associated MSCs engage in reciprocal crosstalk with neutrophils through the IL-6–STAT3–ERK1/2 axis, thereby amplifying the pro-tumor inflammatory niche (39). Similarly, TGF-β signaling is a major mediator of therapeutic resistance in multiple tumor types. In lung cancer, it attenuates anti-PD-1 efficacy by inducing laminin γ2 expression and restricting T-cell infiltration (40). In head and neck cancer, cetuximab treatment activates TGF-β signaling in CAFs, limiting its therapeutic efficacy (41). Pan-cancer analyses have identified a TGF-β-associated ECM gene program that correlates with resistance to PD-1 blockade (42).

Notably, the mTOR signaling pathway plays a critical role in the regulation of MSC function. As an upstream integrator of nutrient availability, growth factors, and cellular energy status, mTOR controls key aspects of MSC biology, including proliferation, self-renewal, differentiation, autophagy, and metabolic adaptation (43, 44). In the context of the TME, aberrant activation of the PI3K/AKT/mTOR axis in tumor-associated MSCs promotes their CAF-like transition, enhances their secretory capacity for tumor-supportive cytokines, and sustains their ability to maintain the CSC niche (45, 46). Moreover, by driving metabolic reprogramming in both MSCs and tumor cells, as evidenced by enhanced glycolysis, glutamine metabolism, and lipid synthesis, the mTOR pathway provides essential metabolic support for cancer stem cell (CSC) stemness and therapeutic resistance (47–49). Therefore, mTOR signaling in MSCs constitutes a fundamental regulatory node that links extracellular cues to intracellular programs that govern stemness and drug resistance.

Collectively, the IL-6/JAK/STAT3 and TGF-β pathways are the core mechanisms through which MSCs enhance tumor stemness and drug resistance. Through these axes, MSCs not only reprogram tumor cells toward self-renewal, survival, and invasiveness but also reshape the surrounding immune and structural contexts to stabilize malignant progression. Wnt/β-catenin serves as a key downstream platform that integrates these stromal inputs. The mTOR pathway, positioned upstream of these cytokine-driven cascades, integrates diverse TME signals to modulate MSC proliferation, differentiation, metabolic adaptation, and autophagy, thereby reinforcing the MSC capacity to support tumor stemness and therapeutic resistance. From a translational perspective, these observations provide a strong rationale for targeting these signaling nodes, including IL-6/IL-6R blockade, JAK/STAT3 inhibition, and disruption of the TGF-β and Wnt pathways, as strategies to disrupt stromal–tumor crosstalk and reverse resistance phenotypes (50). Nevertheless, the biological effects of TGF-β remain strongly context-dependent, highlighting the complexity and therapeutic challenges of targeting these interconnected axes.

### 4.2 Promotes epithelial–mesenchymal plasticity and metastatic potential

A major mechanism by which MSCs facilitate tumor dissemination is the induction and maintenance of EMT and its reversible intermediate states, which are now more precisely referred to as epithelial–mesenchymal plasticity (EMP). Although EMT was originally a physiological program involved in embryogenesis, wound repair, and tissue remodeling, tumor cells have co-opted this process to acquire invasive and metastatic traits (51). This reprogramming is characterized by the dissolution of intercellular junctions, loss of apical–basal polarity, cytoskeletal remodeling, and increased migratory capacity. Importantly, EMT is no longer viewed as a binary epithelial-to-mesenchymal switch but as a dynamic spectrum that includes partial EMT and partial mesenchymal-to-epithelial transition states (52). These hybrid states are increasingly recognized as biologically advantageous because they preserve cellular adaptability, facilitate dissemination, and support survival under therapeutic stress (53). In this context, EMT is closely associated with stemness, dormancy, recurrence, and resistance. This is consistent with the concept of cancer stem cell migration, in which invasive competence and tumor-initiating potential are integrated within the same plastic cellular state (54).

Within this framework, TGF-β remains one of the most potent drivers of EMT in advanced malignancies and represents a major route through which MSCs influence metastatic behavior (**Figure 2B**). Its pro-invasive activity extends beyond canonical SMAD2/3–SMAD4-mediated transcriptional control and involves non-canonical signaling branches, including RAS/MAPK, TAK1–JNK/p38–IKK, and PI3K/AKT, which collectively regulate cytoskeletal organization, cell adhesion, motility, and survival (55). The post-transcriptional regulatory layer is equally important. TGF-β suppresses ESRP1/2 expression and, through ZEB1/2-mediated repression, disrupts epithelial-specific alternative splicing programs, thereby promoting isoform transitions, such as FGFR2-IIIb to FGFR2-IIIc (56–58). These events extend EMT from transcriptional reprogramming to protein isoform remodeling, enabling the coordinated reorganization of adhesion molecules, cytoskeletal components, and motility-associated machinery. This mechanistic breadth helps explain why TGF-β-driven EMT frequently coexists with enhanced stemness and therapeutic resistance. Rather than generating a transient migratory phenotype alone, TGF-β establishes a durable plasticity program that supports residual disease, metastatic seeding, and relapse.

Sustained stromal input enhances MSC-tumor crosstalk. MSCs and their CAF-like derivatives act as persistent sources of TGF-β and chemotactic factors, such as SDF-1/CXCL12, providing both the molecular stimulus for EMT/EMP reprogramming and the directional cues required for tumor cell migration and niche engagement (59). Current evidence increasingly supports the view that the tumor-promoting role of MSCs is best understood not as the delivery of a single effector signal but as the reinforcement of tumor cell fate programs by the surrounding stroma. Experimental evidence from multiple tumor models substantiates this mechanistic framework. In breast cancer, MSCs promote EMT through paracrine TGF-β, and this pathway depends on the ZEB/miR-200 regulatory axis (60). In gastric cancer, MSCs induce EMT through β-catenin-dependent pathways and MMP-16 upregulation, whereas in colorectal cancer, membrane-bound TGF-β on MSC-like cells triggers EMT and enhances circulating tumor cell dissemination. Furthermore, MSC-derived exosomes have been implicated in EMT induction in lung and colorectal cancers (61, 62), and signaling pathways, including MAPK, PI3K/AKT, and Notch, have been identified as downstream mediators in different tumor types. Collectively, these results underscore the heterogeneity of MSC-mediated EMT regulation and confirm the central role of TGF-β signaling. In this regard, TGF-β helps initiate and maintain EMT-associated transcriptional and splicing programs, whereas CXCL12–CXCR4 signaling enhances motility, invasion, and metastatic homing and may further cooperate with TGF-β to stabilize a pro-metastatic microenvironment (63). Through this coordinated input, MSCs couple invasion and dissemination with stemness and resistance, generating tumor cell populations that are more migratory, adaptable, and likely to survive under therapeutic pressure. In addition to their effects on EMT-associated tumor remodeling, MSCs can reinforce metastatic progression and tumor persistence through their immunosuppressive actions within the TME.

### 4.3 MSCs suppress antitumor effector cells

Within the immunosuppressive architecture of the TME, MSCs impair the activity of NK cells and CD8+ cytotoxic T lymphocytes (CTLs) through a coordinated set of soluble, metabolic, and contact-dependent mechanisms. Rather than acting through a single inhibitory pathway, MSCs progressively weaken the essential processes required for effective immune-mediated killing, including proliferation, activation, cytokine production, and cytotoxicity.

In NK cells, MSC-derived mediators, such as PGE2 and IDO, suppress proliferative capacity, reduce IFN-γ secretion, and diminish cytolytic activity, thereby promoting a hyporesponsive or functionally exhausted state (64, 65) (**Figure 2C**). Within tumors, these effects are further reinforced by inhibitory factors such as TGF-β, which disrupt the balance between activating and inhibitory receptor signaling on NK cells. Consequently, even when NK cells are present within the tumor bed, their capacity for degranulation and target cell death is markedly attenuated.

A comparable suppressive logic applies to CTLs, although the dominant mechanisms are particularly evident during their proliferation and effector differentiation. MSCs impose cell cycle and metabolic constraints that restrict the expansion of activated CD8+ T cells and limit the formation of fully competent cytotoxic effectors (66). Nitric oxide (NO)-related pathways contribute to the suppression of T-cell proliferation, whereas inflammatory cues, especially IFN-γ, can license MSCs to adopt a more suppressive phenotype, marked by increased IDO expression (67, 68). By depleting tryptophan and accumulating kynurenine metabolites, this pathway raises the threshold for productive T-cell activation and weakens the generation of cytotoxic responses (69, 70). In this setting, MSCs do not simply inhibit effector cells at the terminal stage of killing; rather, they interfere earlier in the differentiation process, thereby reducing both the quantity and functional quality of tumor-reactive CTLs. This distinction is mechanistically important because impaired expansion and defective effector programming together produce a more durable form of immune suppression than the transient inhibition of cytotoxicity alone.

Beyond these effector-level mechanisms, the mTOR signaling pathway serves as a central hub in MSC-mediated- suppression of NK and CD8^+^ T cells. As a master regulator of metabolism and proliferation, mTOR integrates nutrient and immune signals to control the immune cell fate (71, 72). IDO expressed by MSCs depletes tryptophan, thereby interfering with the tryptophan-sufficiency- signal required for mTOR activation in effector lymphocytes, attenuating their anti-tumor function. For example, recent evidence has linked MSC-mediated suppression to the inhibition of mTOR signaling in NK cells, as demonstrated in the context of gastric cancer, where gastric cancer-derived- MSCs have been shown to reduce NK cell mTOR activity, leading to decreased IFN-γ production and compromised cytolytic capacity (64, 73). In T cells, IDO-mediated tryptophan depletion similarly impairs mTOR activation, resulting in a metabolically quiescent phenotype with reduced glycolysis, increased autophagy, and diminished effector function (74). Collectively, these results establish mTOR signaling as a critical convergence node through which MSCs impair both innate and adaptive immunity, reinforcing an immunosuppressive tumor microenvironment.

The consequences of this suppression extend beyond the direct inhibition of NK cells and CTLs as isolated effectors. NK cells contribute to antitumor immunity not only through immediate cytolysis but also by supporting the recruitment of cDC1 and facilitating cross-presentation, thereby promoting tumor-specific CD8+ T-cell priming and reactivation. When MSCs chronically suppress NK cell function, the TME loses both a rapid innate killing mechanism and an important upstream signal for adaptive immune amplification (75). Simultaneous inhibition of CTLs further compounds this defect, resulting in a tumor niche that is progressively deprived of effective immune surveillance (76). Thus, MSCs suppress antitumor immunity by impairing NK cell and CD8^+^ T-cell function, thereby reducing proliferation, cytokine production, and cytotoxic activity.

### 4.4 MSCs promote the accumulation and functional stabilization of suppressive immune populations

In addition to directly inhibiting effector cells, MSCs actively remodel the tumor immune landscape by organizing the accumulation, differentiation, and functional reinforcement of suppressive immune populations, including Tregs, MDSCs, and TAMs. This process is best understood as a coordinated sequence involving spatial recruitment, lineage polarization, and metabolic reinforcement.

Initially, tumor-associated MSCs contribute to the spatial assembly of an immunosuppressive niche by secreting chemokines that attract suppressive immune cell subsets (77). Among these, the CXCL12/CXCR4 axis and chemokine systems, such as CCL2 and CCL5, play important roles in directing Tregs and myeloid cells toward tumor sites (78). Through this chemotactic program, MSCs not only alter the immune cell composition quantitatively but also establish a cellular framework upon which subsequent immunosuppressive circuits are constructed.

Following recruitment, MSCs further shape the immune cell fate through a combination of soluble immunoregulatory factors and metabolic reprogramming (**Figure 2D**). TGF-β and IL-10 are particularly important in promoting the induction, maintenance, and stability of FOXP3^+^ Tregs, while simultaneously constraining antitumor effector T-cell responses (79). In parallel, inflammatory signals, such as IFN-γ, can license MSCs to acquire a more suppressive phenotype characterized by enhanced IDO expression (80, 81). Activation of the IDO-mediated tryptophan–kynurenine pathway not only impairs effector T-cell function but also favors Treg differentiation and expansion (82). In this respect, MSCs act beyond conventional cytokine secretion; they reshape the biochemical environment, shifting chronic inflammation toward a tolerance-like state. This dual mode of action, which combines soluble mediators with metabolic control, provides a mechanistic basis for the persistent enrichment of suppressive immune populations in the tumor niche.

The suppressive network is then consolidated and amplified through reciprocal interactions centered on MDSCs. As major immunoregulatory effectors within the TME, MDSCs attenuate antitumor immunity through multiple mechanisms, including ARG1-mediated arginine depletion, iNOS- and ROS-dependent stress responses, PD-L1-associated inhibitory signaling, and IDO-related metabolic suppression (83). Simultaneously, they promote the expansion and functional stability of Tregs, thereby establishing self-reinforcing immunosuppressive circuits (84). This network is further strengthened by the broad metabolic architecture of the TME. Adenosine-generating pathways involving CD39/CD73, together with metabolites such as lactate, favor the persistence and activity of Tregs and MDSCs, while rendering effector lymphocytes increasingly vulnerable to nutrient deprivation and metabolic dysfunction (85).

Another major mechanism by which MSCs facilitate tumor immune escape is remodeling of the myeloid compartment, particularly polarization of TAMs toward an M2-like phenotype. Macrophages are among the most abundant immune populations in solid tumors, and their accumulation is frequently associated with poor therapeutic responses and unfavorable clinical outcomes (86). Importantly, TAMs do not exist as a simple M1/M2 binary population; rather, they comprise multiple tissue-resident and monocyte-derived subsets that undergo dynamic spatial and temporal reorganization during tumor progression (87). However, in established malignancies, this heterogeneity generally converges toward immune suppression, tissue remodeling, and metastatic support (88). In this context, tumor-associated MSCs act as persistent stromal instructive cells that bias macrophage differentiation and function toward programs that favor tumor survival.

This polarization is driven by overlapping chemotactic, immunoregulatory, and metabolic signals. MSCs influence monocyte/macrophage recruitment and retention through chemokines and growth factors and subsequently shape macrophage fate through mediators, such as IL-10, TGF-β, PGE2, and IDO (89). Together, these signals suppress proinflammatory macrophage activity while promoting transcriptional and metabolic features associated with an M2-like state, thereby generating TAM populations that are more permissive to angiogenesis, extracellular matrix remodeling, and immune tolerance. In the TME, this process is frequently reinforced by reciprocal interactions with tumor-derived signals, including EVs. For example, in pancreatic cancer, hypoxia-induced tumor exosomes carrying miR-301a-3p promote M2 polarization through the PTEN/PI3Kγ pathway, thereby enhancing metastatic potential (90). Once established, these polarized macrophages become active amplifiers of tumor progression, rather than passive bystanders. M2-like TAM-derived EVs containing miR-365 have been shown to suppress BTG2 and activate FAK/AKT signaling, further promoting pancreatic ductal adenocarcinoma progression. These results illustrate that macrophage polarization is not merely an endpoint of stromal instruction but a mechanism through which new sources of pro-tumor signaling are generated within the microenvironment.

Therefore, the significance of MSC-induced M2-TAM polarization extends beyond the induction of other tumor-promoting cell types. Fundamentally, this reflects the conversion of immune suppression from a transient inhibitory effect to a durable, tissue state. Once macrophages are redirected into an M2-like repair- and tolerance-associated program, they continuously provide anti-inflammatory cytokines, such as IL-10 and TGF-β, support angiogenesis, remodel the extracellular matrix, and reinforce reciprocal signaling with tumor cells and stromal components (91). Through these interactions, immune evasion is coupled with metabolic support, vascular adaptation, and metastasis. Thus, MSC-driven macrophage polarization represents a critical layer in the broader immunosuppressive network of the TME and completes the transition from isolated immune inhibition to a fully established tumor-supportive immune landscape.

Collectively, these observations indicate that MSC-mediated immunosuppression extends beyond the passive release of inhibitory molecules. Instead, MSCs actively orchestrate a multifaceted program that simultaneously suppresses antitumor effector cells and promotes the recruitment, differentiation, and functional reinforcement of suppressive immune cell populations. Through this coordinated action, MSCs establish a durable immune-tolerant state that sustains malignant progression and limits the efficacy of immunotherapy.

### 4.5 Supports tumor angiogenesis

Angiogenesis is a prerequisite for the progression of most solid tumors, from localized growth to sustained expansion and metastatic dissemination. Its initiation is closely linked to hypoxia-driven signaling, particularly the stabilization of HIF-1α, which induces the expression of angiogenesis-related genes, such as vascular endothelial growth factor (VEGF), thereby triggering the angiogenic switch (17, 92). Although this response partially alleviates local oxygen deprivation, it simultaneously provides tumors with renewed perfusion, metabolic support, and structural routes for invasion and dissemination. Within this framework, the pro-tumorigenic role of MSCs, including tumor-associated MSC-like populations, arises not only from their accumulation in the TME but also from their capacity to import a tissue repair-associated proangiogenic program into the neoplastic niche (9, 77). Tumors exploit MSCs as stromal amplifiers of vascular support, converting physiological regenerative functions into mechanisms that sustain malignant progression.

Current evidence indicates that MSCs promote tumor angiogenesis through two major, partially overlapping routes: soluble paracrine signaling and EV-mediated communication (93). At the paracrine level, MSCs secrete various proangiogenic mediators, including VEGF, bFGF/FGF-2, IL-8, PDGF, HGF, TGF-β, and angiopoietins, which collectively enhance endothelial cell survival, migration, proliferation, and tube formation (**Figure 2E**). These effects are further facilitated by matrix remodeling, which lowers the structural barrier for neovessel assembly and allows biochemical angiogenic cues to be translated into functional vascular networks. EV-mediated signaling contributes to another layer of regulation. MSC-derived EVs can deliver proteins, mRNAs, and miRNAs that reprogram angiogenesis-related pathways in recipient endothelial and stromal cells, thereby extending the pro-vascular influence of MSCs beyond direct paracrine exposure. Importantly, hypoxia strengthens this mode of communication by enriching the vesicular cargo linked to VEGF-associated signaling and vascular adaptation, thereby stabilizing a feedback loop in which hypoxia promotes angiogenesis, incomplete reperfusion preserves regional stress, and the resulting microenvironment continues to sustain proangiogenic signaling (94, 95).

However, the proangiogenic capacity of MSCs varies considerably depending on their tissue origin. Bone marrow-derived MSCs (BM-MSCs) are the most extensively studied source and consistently secrete high levels of VEGF and other angiogenic factors, effectively promoting endothelial tube formation and vessel density in multiple tumor models (96, 97).Adipose-derived MSCs (AD-MSCs) exhibit comparable or even higher basal expression of VEGF, bFGF, and PDGF; however, their proangiogenic output is more sensitive to inflammatory priming. Under TNF-α stimulation, AD-MSCs show greater upregulation of IL-8 and monocyte chemoattractant proteins, indirectly enhancing angiogenesis by recruiting myeloid cells (98). Umbilical cord-derived MSCs (UC-MSCs) produce lower baseline levels of VEGF but are particularly responsive to hypoxic preconditioning, leading to robust secretion of HGF and angiopoietin-2 (99, 100). Furthermore, EV cargo differs among MSC sources: BM-MSC-derived EVs are enriched in proangiogenic miRNAs, such as miR-126 and miR-210, whereas AD-MSC-derived EVs carry higher amounts of miR-21 and miR-221, which target endothelial junction proteins (101, 102). These source-dependent differences underscore that the net angiogenic outcome in the TME is not a generic MSC property but reflects the specific origin and activation state of recruited stromal cells.

The vascular contribution of MSCs may extend beyond signaling to include structural participation in vessel maturation and stabilization. Several studies have suggested that MSCs, particularly adipose-derived mesenchymal populations, retain the capacity to acquire pericyte-like features and closely interact with endothelial cells, providing a plausible cellular basis for their involvement in perivascular support and vascular stabilization within tumors (103, 104). Although the extent of this contribution varies substantially across tumor types and microenvironmental contexts, it reinforces the concept that MSC-mediated angiogenesis should be viewed as a coordinated stromal process rather than a single-factor effect. Under hypoxic and inflammatory conditions, MSC-derived proangiogenic signals intersect with vascular-supportive outputs from other stromal and myeloid populations, including tumor-associated macrophages, constructing a vascular niche that supplies nutrients, facilitates metastatic escape, and buffers tumors against therapeutic stress (17, 105). This broader perspective also helps explain why VEGF blockade alone may be insufficient to fully dismantle tumor vascular support in some settings. Therefore, targeting the proangiogenic activity of MSCs and their EVs may represent an important complementary strategy for disrupting stromal contributions to tumor vascularization.

### 4.6 Programming the pre-metastatic niche

Accumulating evidence indicates that metastasis is not a purely stochastic event initiated only after circulating tumor cells reach distant organs, but rather a systemic process prepared in advance by the primary tumor. Before overt metastatic colonization becomes detectable, target organs often undergo measurable histological and molecular alterations, including increased vascular permeability, stromal remodeling, extracellular matrix reorganization, and shifts in local immune composition (106, 107). Collectively, these changes define the pre-metastatic niche (PMN). At this stage, the central event is not the expansion of metastatic lesions, but the conversion of distant tissues from a relatively quiescent ecological state into one that is permissive for tumor cell adhesion, extravasation, survival, and eventual outgrowth (108). Within this conceptual framework, metastasis can be understood as a niche-conditioning process that precedes and facilitates cellular seeding.

At the stromal execution level, MSCs and MSC-like stromal populations are important distal organizers of PMN formation. Their involvement may be conceptualized as a sequential process encompassing recruitment, tissue remodeling, and immune conditioning (109). In response to tumor-derived chemotactic and growth cues, including CXCL12- and platelet-derived growth factor (PDGF)-related signaling, MSCs may accumulate in the perivascular or interstitial compartments of distant organs and establish stromal outposts capable of sustaining paracrine activity. Once present, they reshape local tissue accessibility by secreting mediators, such as CXCL12, CCL2, CCL5, VEGF, FGF, and TGF-β, while matrix-remodeling enzymes and lysyl oxidase-related pathways increase extracellular matrix plasticity and facilitate vascular passage (110, 111) (**Figure 2F**). These changes lower the threshold for subsequent tumor-cell entry. In parallel, MSC-derived chemotactic and immunomodulatory signals promote the recruitment and functional skewing of myeloid populations, thereby shifting the distant immune milieu toward tolerance (112). This aspect of PMN formation is consistent with recent studies emphasizing the premature activation of myeloid- and neutrophil-associated programs, including neutrophil extracellular trap formation, in organs that later become metastatic sites (109). In such settings, stromal networks provide structural and signaling scaffolds that stabilize these changes.

Integrating MSCs into the PMN framework expands their role beyond local production of tumor-supportive factors. A more significant implication is that MSCs directly participate in the structural rewiring of distant organs before the arrival of tumor cells. The perivascular niche is particularly relevant in this context, given its central role in vascular stability, barrier regulation, and paracrine control (113). Increasing attention to pericytes and pericyte-like stromal cells as PMN components provides histological plausibility for the idea that MSCs contribute to distant niche preparation through perivascular interactions or partial differentiation toward pericyte-like phenotypes (114). In parallel, evidence from studies on tumor-associated MSCs suggests that the same stromal properties that promote invasion, angiogenesis, and immune escape at the primary site may also be redeployed during metastatic progression to distal tissues (115). From a translational standpoint, this phase represents a therapeutic window that is distinct from conventional strategies focused on established metastatic lesions. If MSC-mediated recruitment, EV-driven remote signaling, extracellular matrix remodeling, and perivascular niche reorganization are initiated before tumor dissemination is complete, intervention at this stage may disrupt a critical upstream step in metastasis rather than merely responding to its later consequences.

## 5 Potential and mechanisms of MSCs in tumor suppression

In addition to their tumor-promoting roles, MSCs reportedly exert tumor-suppressive effects under specific experimental conditions. These effects are context-dependent and influenced by cellular sources, interaction modes, and microenvironmental factors (**Table 1**).

**Table 1:** Tumor-suppressive effects of MSCs under diverse experimental settings.

Condition type	Specific condition	Result/Implication
Interaction mode	Direct cell-cell contact predominates	Tumor-inhibitory effects are more readily observed, suggesting that some native MSC-mediated antitumor effects depend on contact-mediated signaling rather than solely on paracrine mechanisms.
	Conditioned media, soluble factors, or other paracrine modes predominate	Outcomes become more ambiguous, bidirectional, or may even shift toward tumor support, indicating that the plasticity of the MSC secretome is a major source of biological inconsistency.
MSC tissue source	Menstrual blood-derived MSCs	Induced G0/G1 arrest in HeLa cells, reduced proliferation and invasion, and decreased tumor volume/weight in xenograft models; the effect was linked to MSC-derived TGF-β1 and JNK/p21 activation.
	Amniotic fluid-derived MSCs	Reduced SKOV3 cell viability, accompanied by upregulation of p53/p21 and downregulation of cyclin-related molecules, indicating tumor suppression through cell-cycle reprogramming.
	Wharton’s jelly MSC-derived microvesicles	Inhibit T24 cells, reduce p-Akt, increase cleaved caspase-3
	CD90 low compactness bone-derived MSCs	Activate CD4+/CD8+T cells, reduce Tregs, enhance antitumor activity
Tumor type	Cervical cancer	A cell-cycle arrest-centered inhibitory phenotype appears more readily detectable in this model context.
	Ovarian cancer	Decreased cell viability, altered cell-cycle-related molecules
	Bladder cancer	MSC-derived microvesicles may show a pro-apoptotic, Akt-suppressive antitumor tendency, but this remains EV cargo-dependent.
	Multiple myeloma	The biological output of MSCs/stromal cell-derived EVs is strongly shaped by donor disease status, illustrating how tumor type and its associated milieu can reshape MSC/MSC-EV effects.
Microenvironment/immune context	Immunodeficient model (xenograft)	Direct inhibition of tumor cell growth (cell cycle arrest)
	Immunocompetent model (syngeneic orthotopic)	Partial restoration of antitumor immunity (T cell activation, Treg reduction)
	Combined with inflammation or immune stimulation	Antitumor effects may be strengthened, accompanied by activation of CD4+ and CD8+ T cells and a reduction in Treg cells.
MSC preconditioning/engineering	Unmodified naive MSCs	Limited and unstable antitumor potential, highly condition-dependent
	Engineered MSCs	More predictable and controllable antitumor activity
Experimental design variables	Isolation and expansion protocols	May alter the direction and magnitude of MSC effects and are therefore key contributors to inter-study heterogeneity.
	Passage numbers	May reshape MSC biological output and affect the stability and reproducibility of tumor-suppressive phenotypes.
	Cell doses and MSC:tumor cell ratios	Can influence effect size and contribute to inconsistency across studies.
	Model systems (e.g.xenograft vs syngeneic orthotopic/immunocompetent models)	The observed net phenotype may differ substantially; in particular, immune system integrity directly affects whether immune-rebalancing antitumor effects can emerge.

### 5.1 Defining the boundaries of antitumor effects: model dependence and conditional windows

**Figure 3:**
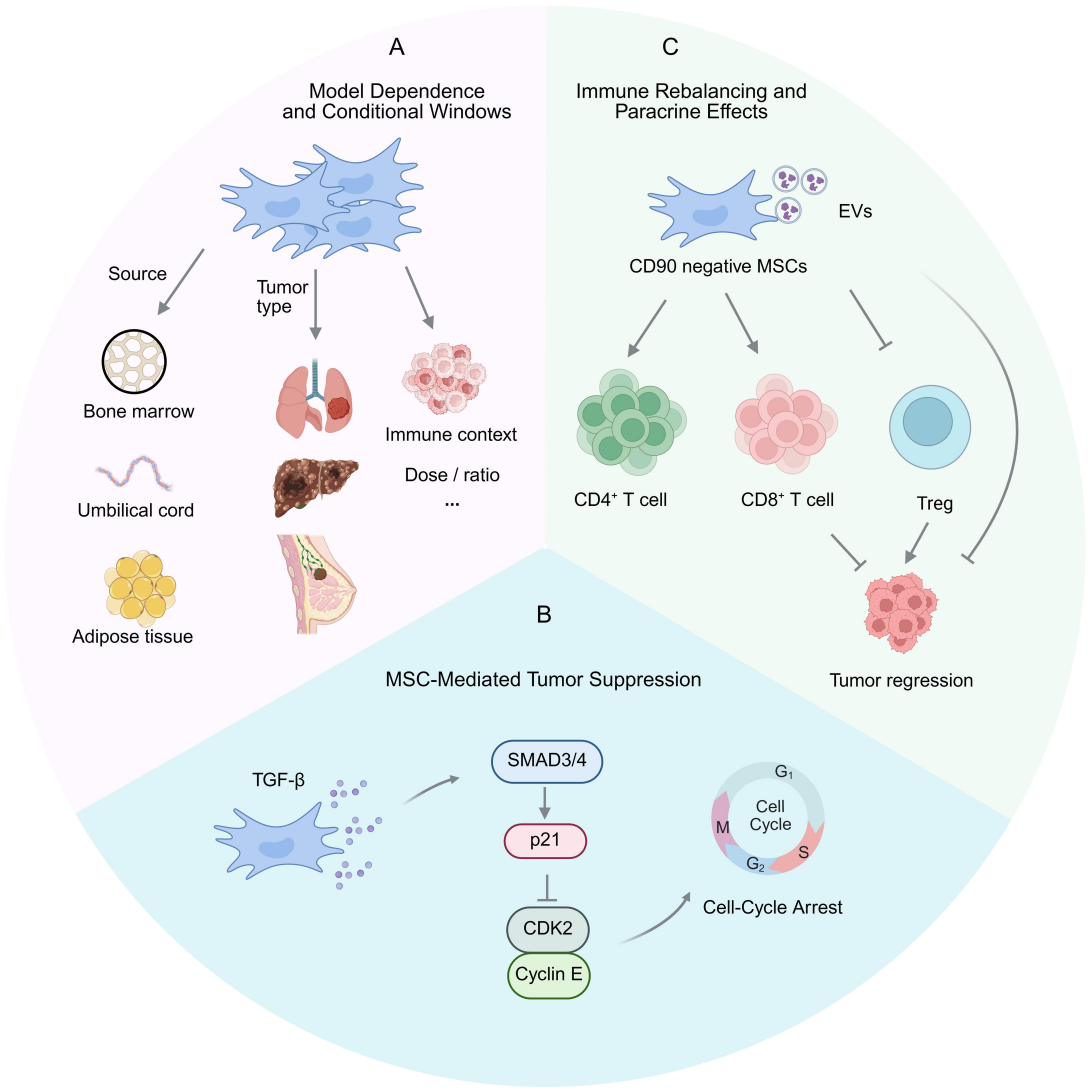
Tumor-suppressive effects of mesenchymal stem cells (MSCs). **(A)** The antitumor effects of MSCs depend on multiple factors, including tissue source (bone marrow, umbilical cord, and adipose tissue), tumor type (lung cancer, hepatocellular carcinoma, and breast cancer), immune context, and cell dose or ratio, which together influence whether tu///tgcemor inhibition occurs. **(B)** MSCs inhibit tumor cell proliferation by inducing cell cycle arrest. MSC-derived TGF-β activates SMAD3/4 signaling, increases p21 expression, and inhibits CDK2/Cyclin E activity, leading to cell cycle arrest. **(C)** MSC-mediated paracrine and immune effects contribute to tumor suppression. MSCs or their extracellular vesicles (EVs), including CD90^-^ MSC subsets, increase CD4^+^ and CD8^+^ T cell activity and reduce regulatory T cells (Tregs), which are associated with tumor regression.

Although the inhibitory effects of MSCs on tumor growth have been documented in selected experimental systems, a rigorous interpretation frames these observations as condition-dependent phenotypes rather than evidence of a universal, intrinsic antitumor property (**Figure 3A**). Recent studies have consistently demonstrated that the direction and magnitude of MSC action are extremely sensitive to multiple experimental and biological variables, including the tissue source, isolation and expansion protocols, passage number, cell dose and ratio, mode of interaction with tumor cells, tumor type, and immunological context (115). Against this backdrop, divergent conclusions across studies are not unexpected and reflect the inherent context-dependence of MSC biology.

A particularly instructive pattern emerged when the modes of interaction were compared. Inhibitory effects are more readily observed in systems with direct cell–cell contact, whereas outcomes often become ambivalent or even shift toward tumor support when interactions are predominantly mediated by conditioned media or other soluble components (116). This pattern aligns with the marked plasticity of the MSC secretome and the differential dependence of tumor cells on contact-mediated versus paracrine signaling. Collectively, these observations establish that the antitumor potential of unmodified MSCs, where it exists, is constrained within specific conditional windows rather than representing a stable or predictable biological property.

### 5.2 Mechanisms of native MSC-mediated tumor suppression: cell cycle arrest

Within the experimentally defined windows, unmodified MSCs exerted tumor-inhibitory activity, particularly in terms of inducing cell cycle arrest (**Figure 3B**). These results suggest that reprogramming along the cell cycle axis may represent a reproducible route through which native MSCs suppress tumor growth.

For instance, in cervical cancer, Liu et al. reported that menstrual blood-derived MSCs induced G0/G1 arrest and reduced the proliferation and invasion of HeLa cells, accompanied by decreased tumor volume and weight in xenograft models (117). Mechanistically, this effect was linked to MSC-derived TGF-β1 and the activation of the JNK/p21 pathway in tumor cells. Similar results have been reported in ovarian cancer models, in which amniotic fluid-derived MSCs reduced SKOV3 cell viability, accompanied by the upregulation of p53/p21 and downregulation of cyclin-related molecules (118). These observations collectively indicate that the disruption of cell cycle progression and survival signaling are the core mechanisms underlying MSC-mediated tumor suppression in permissive experimental contexts.

However, these inhibitory effects cannot be generalized to all MSC populations. Even within the same tumor type, MSCs from different tissue sources may engage in distinct pathways and produce different net outcomes (119). Reviews in gynecologic oncology have similarly indicated that MSCs and their conditioned media can suppress tumor growth in certain models, although the magnitude and characteristics of these effects are strongly influenced by cell origin and experimental design (21). A critical observation is that the net phenotype may shift when the dominant mode of interaction transitions from direct to secretion-dependent communication. Such dynamic changes likely represent a key source of inconsistency in the reproducibility and standardization of results related to native MSC-mediated tumor suppression, underscoring the precarious nature of relying on unmodified MSCs for antitumor effects (120).

### 5.3 Conditional nature of immune rebalancing and paracrine effects: potential antitumor activity under permissive contexts

The conditional nature of MSC-mediated tumor suppression becomes even more apparent when attention is shifted from direct cellular interactions to conditioned media or EVs, where inconsistencies in biological outcomes are often the most pronounced (**Figure 3C**). This domain also warrants caution in interpreting antitumor claims, as MSC paracrine output is highly plastic and context dependent.

A representative example is a report showing that microvesicles derived from Wharton’s jelly MSCs inhibited bladder cancer T24 cells, accompanied by reduced p-Akt levels and increased cleaved caspase-3. This suggests that certain EV cargo compositions can increase the susceptibility of tumor cells to apoptosis. However, EV content is highly dependent on the donor status and disease context (121). In multiple myeloma, exosomes derived from bone marrow stromal cells differ substantially from those of healthy controls in terms of miRNA and protein cargo, and these differences are associated with markedly distinct tumor biological effects (122). These results underscore that the antitumor potential of MSC-derived EVs cannot be inferred from the label “MSC-EV” alone but must be interpreted in light of the cellular source, activation state, and microenvironmental conditioning.

A similar principle applies to immune regulation. In immunocompetent settings, the net effect of native MSCs may be reweighted by the surrounding immune network, potentially shifting from the predominantly immunosuppressive phenotype observed in many tumor contexts to partial immune rebalancing. In an isogenic orthotopic ovarian cancer model, Zeng et al. observed that CD90 low compactness bone-derived MSCs exerted antitumor activity, which was further enhanced in combination with immune stimulation, accompanied by the activation of CD4+ and CD8+ T cells and a reduction in regulatory T cells (123). These observations suggest that in permissive inflammatory or immune contexts, native MSCs may contribute to the partial restoration of antitumor immunity rather than exclusively reinforcing immune suppression.

Collectively, the available evidence supports the existence of tumor-suppressive effects mediated by native MSCs and their extracellular products under defined experimental conditions (124, 125). However, the direction, stability, and reproducibility of these effects remain highly variable and are strongly influenced by the tissue source, culture history, interaction mode, and microenvironmental context (126). The major implication is that MSCs do not possess stable intrinsic antitumor properties; however, their biological output can shift toward growth restraint, apoptosis sensitization, or partial immune activation within certain conditional windows (127).

This result has two significant implications. First, it underscores the need for rigorous experimental reporting and interpretation that acknowledge the context-dependent nature of MSC biology, moving away from sweeping generalizations about “antitumor MSCs.” Second, and more importantly, it provides a rationale for controllable engineering approaches that harness the native homing properties of MSCs while redirecting their functional output toward more predictable and therapeutically exploitable antitumor activity. Rather than relying on the intrinsic potential of unmodified MSCs, the field is increasingly moving toward the design of MSC-based therapeutic platforms that deliver targeted payloads or respond to engineered regulatory circuits (128).

MSCs lack an intrinsic pro-tumor or anti-tumor identity; their ultimate function is dictated by local signals encountered within the tumor microenvironment (TME), resulting in a distinct dual role. In most solid tumors, hypoxia, inflammation, and tumor-derived- cues drive MSCs toward a tumor-promoting- phenotype. They secrete IL-6- and TGF-β- to enhance tumor cell stemness and drug resistance, induce epithelial-mesenchymal- plasticity to promote invasion and metastasis, suppress NK and CD8^+^ T cell function, recruit regulatory T cells, myeloid-derived- suppressor cells, and M2-type- macrophages to form an immunosuppressive network, and support angiogenesis and pre-metastatic niche formation. However, under specific experimental conditions, MSCs can exert anti-tumor effects. Certain MSC sources induce cell cycle arrest via the TGF-β1/JNK/p21 or p53/p21 pathways, or partially restore effector T cell function while reducing regulatory T cells in immunocompetent models. These anti-tumor effects are strictly condition-dependent-; they typically require direct cell-cell- contact and are strongly influenced by MSC tissue origin, tumor type, and microenvironmental context, often diminishing or even switching to tumor promotion in paracrine-dominant- settings. Thus, the dual role of MSCs in tumors reflects their phenotypic plasticity across different microenvironments, providing a theoretical basis for engineering MSCs for controllable anti-tumor therapies.

## 6 Turning enemies into allies: armed MSCs as antitumor weapons

### 6.1 Overexpression of antitumor effectors through genetic engineering

**Figure 4:**
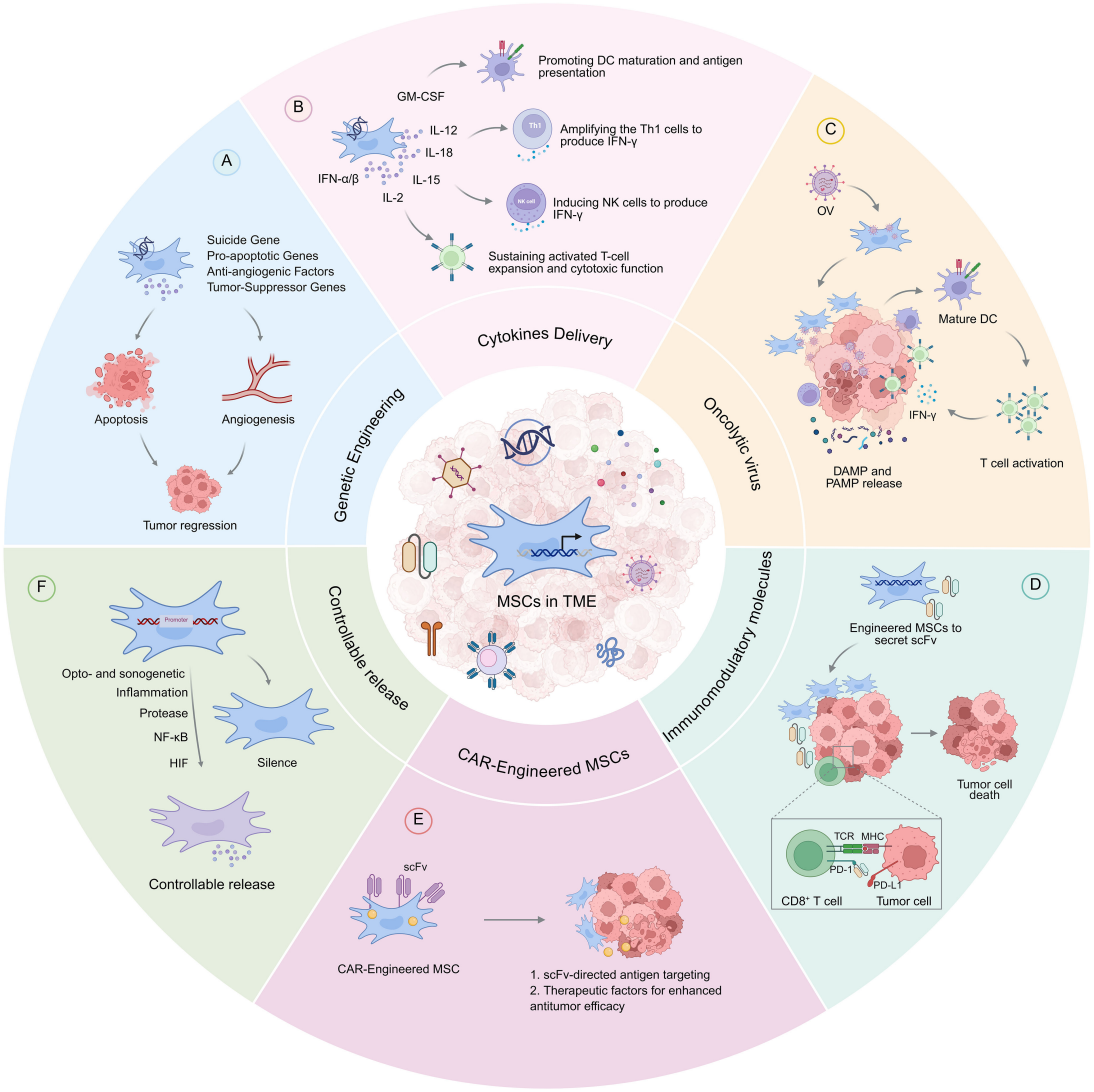
Engineered mesenchymal stem cell (MSC)-based strategies for tumor-targeted therapy. **(A)** Genetically engineered MSCs deliver antitumor effectors, including suicide genes, pro-apoptotic genes, anti-angiogenic factors, and tumor suppressor genes, leading to tumor cell apoptosis, reduced angiogenesis, and tumor regression. **(B)** MSCs engineered to secrete cytokines, such as interleukin-2 (IL-2), IL-15, IL-12, IL-18, granulocyte-macrophage colony-stimulating factor (GM-CSF), and interferon-alpha and interferon-beta (IFN-α/β), promote dendritic cell maturation and antigen presentation, enhance Th1 responses, activate NK cells to produce interferon-gamma (IFN-γ), and support T-cell expansion and cytotoxic function. **(C)** MSCs act as carriers of oncolytic viruses (OVs). Viral infections induce tumor cell lysis and the release of damage-associated molecular patterns (DAMPs) and pathogen-associated molecular patterns (PAMPs), which promote dendritic cell maturation and T-cell activation. **(D)** Engineered MSCs deliver immunomodulatory molecules, including secretable scFv targeting immune checkpoints, thereby disrupting the inhibitory PD-1/PD-L1 interaction between CD8^+^ T cells and tumor cells, restoring T-cell receptor (TCR)-mediated T cell activation, and promoting tumor cell death. **(E)** CAR-engineered MSCs improve tumor targeting and retention, allowing for antigen-directed delivery of therapeutic factors and enhanced antitumor effects. **(F)** Synthetic biology-based systems enable the controlled release of therapeutic cargo from MSCs in response to tumor-associated signals or external stimuli, thereby improving the control over therapeutic activity.

Genetic engineering is a key strategy for converting MSCs from context-dependent stromal cells into programmable antitumor platforms. Rather than relying on the inherently variable biological effects of naïve MSCs, engineered MSCs can be endowed with a defined therapeutic cargo and exploit their tumor-tropic behavior to achieve localized, sustained, and mechanistically controlled antitumor activity (129, 130). In this setting, MSCs function not only as passive carriers but also as cellular factories that deliver and produce therapeutic effectors directly within the TME, thereby increasing local bioavailability while potentially reducing systemic toxicity. Based on the nature of the introduced effector molecules, current MSC-based genetic engineering strategies can be broadly categorized into four major groups: suicide gene systems that generate cytotoxic metabolites *in situ*, pro-apoptotic genes that directly induce tumor cell death, anti-angiogenic factors that disrupt tumor vascular support, and tumor suppressor genes that restore inhibitory signaling in malignant tissues (**Figure 4A**). The following subsections discuss these four categories and illustrate how the enforced expression of defined antitumor effectors expands the therapeutic scope of MSC-based cancer therapies.

#### 6.1.1 Suicide gene

Among the genetically engineered antitumor effectors introduced into MSCs, suicide gene systems represent the most extensively studied and conceptually mature strategies. Their therapeutic principle is based on exploiting MSC tumor tropism to deliver prodrug-converting enzymes into the TME, where the subsequent systemic administration of the corresponding prodrug generates cytotoxic metabolites locally. Suicide gene therapy combines the spatial selectivity of MSC homing with the pharmacological controllability of enzyme/prodrug systems, thereby enhancing local tumor killing and limiting off-target toxicity (131). Within this category, the herpes simplex virus thymidine kinase/ganciclovir (HSV-TK/GCV) and cytosine deaminase/5-fluorocytosine (CD/5-FC) systems, particularly the yeast cytosine deaminase–uracil phosphoribosyltransferase (yCD: UPRT)/5-FC variant, have been the most extensively investigated platforms.

The HSV-TK/GCV system remains one of the best-characterized suicide gene approaches for MSC-based therapies (132, 133). In this setting, HSV-TK expressed by engineered MSCs phosphorylates ganciclovir into toxic metabolites, leading to the death of transduced cells and bystander-mediated killing of adjacent tumor cells (134). This feature is particularly relevant in glioblastomas, where diffuse infiltration limits the effectiveness of conventional local therapies, making cell-based local delivery attractive. In contrast, the CD/5-FC system relies on the intratumoral conversion of the relatively nontoxic prodrug 5-fluorocytosine into 5-fluorouracil, which is especially advantageous for brain tumors because 5-FC can cross the blood-brain barrier (135, 136). Preclinical studies on melanoma, prostate cancer, ovarian cancer, and intracranial glioma models have shown that MSC-mediated delivery of CD/5-FC or yCD: UPRT/5-FC effectively suppresses tumor growth and, in some settings, induces marked tumor regression (137, 138). Moreover, both suicide gene platforms have demonstrated greater therapeutic value when incorporated into combination strategies. Recent studies have suggested that coupling suicide genes with immunomodulatory elements may further broaden their antitumor potential.

Despite these encouraging results, MSC-based suicide gene therapy is strongly influenced by tumor context and biological heterogeneity. The therapeutic efficacy of both the TK/GCV and CD/5-FC systems depends not only on transgene expression but also on factors, such as the efficiency of intercellular communication, the extent of bystander killing, and the intrinsic drug-response properties of tumor cells, all of which may contribute to variable outcomes across models (131, 139). Moreover, because MSCs exhibit context-dependent functional plasticity, the clinical translation of engineered MSC products requires rigorous optimization of the cell source, delivery route, treatment timing, and safety control. Nevertheless, suicide gene systems have provided important proof-of-concept and early translational evidence for MSC-based antitumor engineering, particularly in glioblastomas. Collectively, these results establish HSV-TK/GCV and CD/5-FC as foundational examples of how genetic modifications can endow MSCs with defined cytotoxic effector functions, thereby laying a conceptual basis for the subsequent development of pro-apoptotic, anti-angiogenic, and tumor-suppressive MSC platforms.

#### 6.1.2 Pro-apoptotic genes

Among the antitumor effectors introduced into MSCs, pro-apoptotic ligands and cytokines are particularly attractive because they convert tumor-tropic cells into localized sources of death signaling. The best-established example is TRAIL, which binds to DR4 and DR5, promotes the assembly of the death-inducing signaling complex, and activates initiator caspases to trigger apoptosis (140). In engineered MSC systems, the advantage of TRAIL delivery is less in simple ligand overexpression than in sustained intratumoral exposure, thereby increasing the likelihood of overcoming incomplete receptor engagement and resistance to apoptosis. This spatially restricted mode of delivery is mechanistically important because tumor cell responsiveness to TRAIL is often curtailed by anti-apoptotic regulators, such as c-FLIP and members of the BCL-2 family. Therefore, MSC-based delivery provides a means of maintaining local apoptotic pressure within the tumor niche while avoiding the rapid clearance and limited persistence associated with systemic administration.

FasL represents a related but less fully developed death receptor strategy. By engaging Fas (CD95), FasL activates the extrinsic apoptotic pathway in susceptible target cells, thereby offering a second route for MSC-mediated induction of tumor cell death (141). Direct oncological evidence remains limited compared to that for TRAIL; however, the available studies support the biological plausibility of this approach (142, 143). In multiple myeloma, MSCs with high FasL expression suppress tumor growth, reduce metastasis and bone destruction, and increase tumor cell apoptosis, whereas FasL-deficient MSCs lose this inhibitory activity (144). Complementary support comes from vesicle-based studies, in which adipose-derived MSC microvesicles containing Fas/FasL and other pro-apoptotic proteins inhibit the growth of primary ovarian cancer cells. Recent studies suggest that MSC-derived apoptotic vesicles can retain biologically active membrane-associated death signaling, although direct cancer-specific evidence for functionally active FasL in engineered MSC-derived ApoVs remains limited (145, 146). Collectively, these observations suggest that FasL is a credible pro-apoptotic payload for MSC-based engineering, although its development as a tumor-directed platform is still in its early stages and is less standardized than that of TRAIL.

Type I interferons, particularly IFN-β, broaden this category beyond receptor-triggered apoptosis. Through IFNAR-mediated JAK/STAT activation, IFN-β induces interferon-stimulated genes that suppress proliferation, enhance susceptibility to apoptosis, and reshape tumor-cell interactions with the immune microenvironment (147). In MSC-based systems, this activity extends beyond the direct growth inhibition. Recent studies on glioblastoma have shown that engineered MSCs secreting IFN-β can induce tumor-cell apoptosis and, when paired with local PD-1 blockade, enhance T-cell activation and T-cell-mediated tumor killing (148). In this sense, TRAIL and FasL primarily function as locally sustained death triggers, whereas IFN-β provides a broader antitumor program that integrates pro-apoptotic, antiproliferative, and immune-reprogramming effects (140, 143). Collectively, these strategies illustrate the central logic of apoptotic MSC engineering, and therapeutic benefits arise not from indiscriminate overexpression of cytotoxic molecules but from confining mechanistically distinct pro-death signals to the tumor site, where they can act with greater persistence, selectivity, and biological relevance.

#### 6.1.3 Anti-angiogenic effectors

While TRAIL- and IFN-β-based strategies primarily target tumor-cell survival and apoptotic susceptibility, the antitumor utility of engineered MSCs can also be redirected toward the vascular niche that sustains malignant growth. In many solid tumors, therapeutic failure reflects not only cell-intrinsic resistance programs but also the persistence of a structurally abnormal yet functionally supportive vasculature that maintains nutrient delivery, facilitates adaptation to hypoxic stress, and creates routes for invasion and dissemination (149). This has shifted the focus of MSC engineering from direct cytotoxic signaling to the local disruption of tumor-supportive architecture. In this context, anti-angiogenic modification is better understood as a niche-directed intervention than as a simple inhibition of a single growth factor pathway because tumor angiogenesis is regulated by interconnected VEGF, FGF, PDGF, and angiopoietin networks that influence endothelial proliferation, vascular permeability, perfusion heterogeneity, and the formation of hypoxic territories linked to drug resistance and tumor evolution (150).

Within this category, soluble VEGF receptors, particularly soluble VEGFR-1 (sVEGFR-1/sFlt-1), are among the clearest MSC-based anti-angiogenic payloads (151). Mechanistically, sFlt-1 acts as a decoy receptor, sequestering VEGF and blunting downstream VEGFR signaling, thereby limiting endothelial activation, neovascularization, and tumor-associated vascular support (152). In preclinical models, systemically delivered BMSCs engineered to express sFlt-1 preferentially home to tumor sites, reduce lung metastases, and prolong survival through combined anti-angiogenic and pro-apoptotic effects (153). Subsequent studies on hepatocellular carcinoma have further shown that MSC-sFlt-1 can be integrated into combination strategies, paired with continuous low-dose doxorubicin, to enhance apoptosis, suppress microvessel density, and drive xenograft remission *in vivo* (154). These results are conceptually important because they indicate that MSC-based anti-angiogenic therapy is not limited to starving tumors of blood supply but can also sensitize the vascular niche and surrounding tumor tissue to additional therapeutic pressure.

Endostatin and angiostatin extend this strategy beyond sequestration. Endostatin, an endogenous inhibitor of angiogenesis derived from collagen XVIII, is widely recognized as a vascular inhibitory protein with comparatively favorable safety characteristics. MSC-mediated delivery has been developed to overcome the limited persistence of soluble protein administration. In ovarian cancer models, placenta-derived MSCs engineered to express endostatin retain tumor tropism, reduce tumor volume without obvious systemic toxicity, decrease blood sprouting and tumor cell proliferation, and increase apoptosis, supporting the view that endostatin-expressing MSCs can suppress both neovessel formation and the broader tumor-supportive consequences of aberrant vascularization (21, 155, 156). Angiostatin, a plasminogen-derived endogenous angiogenesis inhibitor, belongs to the same conceptual class of vascular-restrictive payloads and is mechanistically relevant because it suppresses endothelial proliferation and migration. However, compared with sFlt-1 and endostatin, the direct development of MSC-delivered transgenes appears less extensively documented in the literature (149, 157). Collectively, these observations suggest that the value of anti-angiogenic MSC engineering lies not only in antagonizing VEGF but also in locally perturbing the vascular maturation–perfusion axis that supports tumor persistence, metastatic competence, and treatment tolerance, thereby creating a more favorable therapeutic window for combination with cytotoxic, immune, or gene-based interventions.

#### 6.1.4 Tumor-suppressive factors

Genetic engineering can extend MSC-based anticancer therapy beyond cytotoxic and anti-angiogenic modules by enabling the localized delivery of tumor-suppressive factors, such as PTEN and p53 (158). This strategy is mechanistically distinct from enzyme/prodrug or death-receptor systems because its purpose is to re-establish intracellular restraints that are commonly disabled during malignant progression. PTEN is particularly relevant in this context because it antagonizes PI3K-AKT-mTOR signaling, thereby constraining proliferation, survival, migration, and treatment resistance, while regulating stem cell proliferation, differentiation, metabolism, and genomic stability (159). However, PTEN-engineered MSCs should be regarded as an early proof-of-concept platform rather than a broadly validated antitumor modality because efficacy data across tumor types, delivery settings, and clinically relevant models remain sparse (160, 161). The rationale for incorporating p53 is equally strong, as p53 coordinates cell cycle arrest, DNA repair, apoptosis, senescence, metabolism, and immune regulation, and TP53 remains the most frequently altered tumor suppressor axis in human cancers. Recent therapeutic literature has further reinforced the translational appeal of p53 restoration, including nucleic acid-based approaches designed to recover wild-type p53 activity in tumors that have lost or inactivated this pathway (162). Within the MSC field, a more immediate lesson comes from carrier biology itself: mesenchymal loss of p53 alters stem cell properties and is sufficient to drive aggressive undifferentiated soft-tissue sarcomas, indicating that p53 integrity must be preserved as a prerequisite for any MSC platform intended to function as a tumor-suppressive vehicle (163). Accordingly, PTEN- and p53-oriented engineering currently represents a promising but underdeveloped branch of MSC-based anticancer design. Its advancement depends on tumor-restricted expression systems, stronger efficacy data, and rigorous control of carrier-derived pro-tumorigenic risk.

Overall, the therapeutic value of MSC-based genetic engineering lies not only in overexpressing antitumor molecules but also in using MSCs as tumor-directed delivery vehicles to concentrate these effectors at the disease site. By exploiting the intrinsic tumor-homing and stromal-retention properties of MSCs, therapeutic signals can be deployed where needed, thereby increasing local bioavailability while limiting systemic exposure and off-target toxicity. In this sense, the significance of MSC engineering extends beyond the quantity of the expressed molecule to the spatial precision of its delivery, allowing high local activity, prolonged intratumoral retention, and a more favorable therapeutic index.

### 6.2 MSC-based delivery of immunostimulatory cytokines for local antitumor immune activation

Rational engineering of MSCs as immunotherapeutic platforms requires a clear understanding of the cytokines that can be effectively deployed within the TME and the mechanisms by which they reinforce antitumor immunity. The following subsections examine four classes of immunostimulatory molecules (**Figure 4B**), each targeting distinct yet partially overlapping immune effector and accessory populations.

### 6.2.1 IL-2 and IL-15 for reinforcing CD8+ T cells and NK cells immunity

In cytokine-based MSC engineering, IL-2 and IL-15 are particularly relevant due to their action on CD8+ T cells and natural killer (NK) cells, which are central to antitumor immunity (164, 165). However, their therapeutic application is constrained by poor pharmacokinetics, limited tumor biodistribution, and off-target toxicity (166). MSCs offer a promising delivery platform by leveraging their tumor-homing and stromal retention properties to concentrate immunostimulatory signals within the TME.

IL-2 is essential for T cell expansion and function, but its systemic use is limited by regulatory T cell activation and peripheral toxicity. Recent engineering efforts have focused on tumor-restricted delivery and receptor retuning to enhance effector lymphocyte stimulation while reducing systemic side effects (167, 168). This is particularly relevant in immune checkpoint resistance, where insufficient functional CD8^+^ TILs pose a major bottleneck. A recent preclinical study demonstrated that MSCs delivering an IL-2 mutein dimer reinvigorated pre-existing CD8^+^ TILs, overcame resistance to immune checkpoint blockade, and achieved antitumor activity without overt toxicity (169). Thus, MSC-mediated IL-2 delivery restores local IL-2 signaling in the tumor niche, promoting CD8^+^ T cell expansion and functional rescue without systemic amplification.

IL-15, through IL-15Rα-dependent trans-presentation, preferentially supports NK cells and memory-like CD8^+^ T cells without expanding regulatory T cells (165, 170). However, its short half-life and dose-limiting toxicity necessitate sustained local administration. While recent strategies combining local IL-15 delivery with PD-L1 blockade have enhanced antitumor immunity in poorly inflamed tumors, emerging MSC-based designs further improve tumor localization and provide sustained support to NK cells (171, 172). In MSC engineering, IL-15 complements IL-2 by reinforcing intratumoral cytotoxic competence and immune persistence while extending support to NK cell-mediated tumor control.

#### 6.2.2 IL-12 and IL-18 in Th1- and NK-oriented antitumor immunity

Unlike IL-2 and IL-15, which support cytotoxic lymphocyte persistence, IL-12 and IL-18 induce type 1 immune responses within the TME (173, 174). IL-12 promotes IFN-γ production and enhances T/NK cell activity via STAT4 signaling (175), but its systemic toxicity necessitates local delivery (176). MSC-based delivery concentrates IL-12 within tumors, enhancing local immune activation while reducing peripheral exposure. Recent studies confirm this approach: IL-12-expressing MSCs increased CD4^+^ T and NK cell infiltration, reduced Tregs, and complemented anti-PD-1 therapy in glioblastoma models (177, 178); similar antitumor effects were observed in lymphoma models (179). Additionally, IL-12/15/18 stimulation induces metabolic reprogramming in memory-like NK cells, supporting long-term functional competence (180, 181).

IL-18 promotes IFN-γ production via MyD88 signaling and synergizes with IL-12 to strengthen type 1 immunity (182). However, its context-dependent pro-tumorigenic effects (e.g., in angiogenesis and metastasis) require controlled dosing and spatial restriction (183). Engineered IL-18 formats with improved stability and tumor selectivity have addressed these limitations (184–186). In glioblastoma models, MSCs co-expressing yeast cytosine deaminase and IL18-Fc achieved enhanced therapeutic benefits, supporting the feasibility of combining cytokine engineering with cell-based delivery (135, 177).

Collectively, these results suggest that IL-12 and IL-18 can be understood as locally deployed type 1 immune modulators whose principal therapeutic value lies in strengthening Th1- and NK-oriented antitumor immunity at the tumor site, thereby improving immune recruitment, functional activation, and therapeutic selectivity of cytokine-based interventions.

#### 6.2.3 GM-CSF promotes dendritic-cell maturation and antigen presentation

Unlike cytokines that directly enhance T/NK cell function, GM-CSF acts at an earlier stage by regulating dendritic cell (DC) differentiation, maturation, and activation. DCs are principal antigen-presenting cells that link innate and adaptive immunity, playing a decisive role in tumor antigen uptake, cross-presentation, and naïve T cell priming (187, 188). Accordingly, the immunotherapeutic significance of GM-CSF lies less in its conventional classification as a myeloid growth factor than in its capacity to shift tumor-associated antigen presentation from a functionally insufficient state to a more immunostimulatory state (189, 190).

GM-CSF enhances DC recruitment, survival, maturation, and cross-presentation, thereby facilitating tumor-specific CD8+ T cell responses (191). Local GM-CSF delivery increases DC and antigen-specific T cell accumulation within tumors (192). Recent studies show that GM-CSF-overexpressing MSCs induce the differentiation of murine monocytes into DCs and promote a proinflammatory phenotype (193). However, MSCs secreted GM-CSF has also been implicated in promoting tumor cell proliferation, invasion, and transendothelial migration (194). Therefore, the therapeutic value of GM-CSF depends on restoring local antigen-presentation networks and increasing adaptive immune initiation efficiency, while mitigating its potential pro-tumorigenic effects through strategies such as localized delivery and context-dependent engineering.

#### 6.2.4 Type I interferons (IFN-α/β) as cytokines targeting multiple immune-cell populations

In contrast to cytokines that act on defined effector compartments, type I interferons (IFN-α/β) broadly influence immune populations (195). Through JAK1–TYK2–STAT1/2 signaling, they enhance DC activation and cross-presentation, CD8^+^ T cell priming, NK function, and modulate myeloid states (196). IFN-α is primarily produced by pDCs promoting type 1 immunity, whereas IFN-β is produced by various cells enabling broader TME signaling (197, 198). The biological consequences of IFN-α/β signaling are highly context-dependent. Acute, restricted activation enhances antigen presentation and effector cell recruitment, whereas chronic low-level signaling may induce immunosuppressive mediators such as PD-L1 and CCL22 (199). Therefore, therapeutic benefit requires controlled signaling strength, duration, and distribution.

These considerations have shifted current therapeutic strategies toward the spatially controlled delivery of IFN-α/β rather than diffuse systemic administration. One approach directs IFN-α/β specifically toward dendritic cell subsets, particularly cDC1, to improve cross-presentation and CD8^+^ T cell priming while mitigating the short half-life and systemic toxicity that have historically limited interferon therapy (200). This principle has been extended to engineered cellular delivery systems. In glioblastoma, MSCs engineered to secrete IFN-β induced tumor cell apoptosis and enhance T cell activation and cytotoxicity when combined with PD-1 blockade. Moreover, IFN-α/β increase MHC class I expression on tumor cells, promote CXCL10-dependent recruitment of effector T cells, and shift macrophages toward a more inflammatory phenotype (201). Compared with conventional systemic interferon therapy, these controlled delivery strategies offer a more balanced approach to efficacy and tolerability.

By exploiting the intrinsic tumor-homing and stromal-retention properties of MSCs, cytokines such as IL-2, IL-15, IL-12, IL-18, and IFN-α/β can be concentrated within the TME, enhancing local immune activation while reducing systemic toxicity that has long limited conventional cytokine therapy (10). Engineered MSCs thus overcome several persistent bottlenecks of cytokine-based treatments, including short half-life, narrow therapeutic window, and diffuse off-target exposure (128). However, cytokine delivery primarily strengthens immune activation and effector cell function, and its capacity to reverse the established immunosuppressive architecture remains incomplete (189). This limitation provides a clear rationale for the next level of MSC-based engineering, in which MSCs are used to carry oncolytic viruses that can directly lyse tumor cells, initiate inflammatory danger signaling, and profoundly disrupt the immunosuppressive TME. Within this framework, the transition from cytokine delivery to oncolytic viral loading represents a shift from immune reinforcement alone to a more comprehensive strategy of immune microenvironment remodeling.

### 6.3 Loading oncolytic viruses to disrupt the immunosuppressive TME

Loading oncolytic viruses (OVs) onto MSCs combines two complementary biological properties: the capacity of OVs to propagate within tumors through sequential cycles of replication and lysis, and the intrinsic ability of MSCs to migrate toward tumor- and inflammation-associated niches and persist within stromal compartments (202, 203)(**Figure 4C**). This strategy addresses several limitations of systemic OV administration, including rapid circulatory clearance, first-pass sequestration, and immune pressure neutralization. By functioning as cellular carriers, MSCs can shield OVs from premature inactivation, facilitate delivery to the tumor site, and provide a transient permissive environment for viral amplification before local release in certain settings (204). In ovarian cancer, MSC-based delivery of recombinant oncolytic adenoviruses, such as Ad5/3 and D24RGD, has shown greater antitumor efficacy than free viruses in orthotopic and peritoneal models, while preserving activity under antibody-mediated neutralization (118, 203). Similar carrier-assisted strategies have been explored in glioblastomas, where MSC-mediated delivery improves viral dissemination and supports early clinical translation (205).

The therapeutic effects of MSC-OV platforms extend beyond their transport. Once released within the tumor, OVs infect malignant cells and induce direct oncolysis and immunogenic cell death, leading to the release of tumor-associated antigens and danger signals, including ATP, HMGB1, and calreticulin (206, 207). Viral infection also activates innate immune pathways, including cGAS-STING-dependent type I interferon signaling, and promotes inflammatory chemokine production, dendritic cell maturation, antigen cross-presentation, and subsequent CD8^+^ T cell priming (208) (**Figure 4C**). Through these coordinated events, OV therapy can convert poorly infiltrated or immunologically quiescent tumors into inflamed lesions enriched with effector immune cells. This capacity to couple local tumor destruction with immune activation distinguishes OV-based delivery from cytokine-only strategies and is particularly relevant for tumors characterized by limited baseline immunogenicity.

Another contribution of OV therapy is its ability to remodel suppressive stromal and myeloid programs. OV infection can repolarize tumor-associated macrophages from an M2-like state to an M1-like phenotype by suppressing STAT6- and STAT3-associated immunosuppressive pathways and activating inflammatory signaling downstream of DAMP- and PAMP-sensing receptors (209). This transition is accompanied by increased expression of inflammatory mediators, enhanced antigen-presenting and phagocytic functions, and improved support for NK cell and T cell activity, thereby weakening the suppressive networks within the TME. Emerging evidence suggests that GM-CSF-armed oncolytic herpes simplex virus (oHSV) enhances the response to PD-1 blockade and strengthens NK cell-mediated cytotoxicity (210, 211). In parallel, other microenvironment-disruptive approaches can be incorporated into this framework, including viral platforms armed with extracellular matrix-degrading enzymes, such as hyaluronidase, which improve intratumoral spread and facilitate immune cell access in structurally restrictive tumors. Available evidence supports the view that MSC-mediated OV delivery is a locally amplified therapeutic strategy that integrates protected viral transport, intratumoral self-amplification, direct tumor lysis, and a broader disruption of the immunosuppressive microenvironment.

### 6.4 Delivery of immunomodulatory molecules, particularly immune checkpoint-blocking formats

Engineered MSCs can adapt beyond cytokines and oncolytic viruses to deliver immunomodulatory molecules that alleviate the inhibitory signaling within the TME. Current direct evidence has concentrated on PD-1/PD-L1-directed blocking formats, particularly secretable scFv and virally encoded PD-L1 blockers, rather than conventional full-length monoclonal antibodies (147, 212). A representative example has been reported in glioblastoma, where MSCs were engineered to express a secreted single-chain variable fragment targeting PD-1. In this model, MSC-derived scFv-PD1 enhanced T-cell activation and T-cell-mediated tumor killing (**Figure 4D**), and local administration of dual-engineered MSCs reduced tumor burden, prolonged survival, and reshaped the postoperative TME following resection (147). These results support the concept that MSC platforms can localize checkpoint-blocking activity within tumor tissues while coupling it to a second immunomodulatory signal of complementary function.

A related strategy uses MSCs to deliver combinatorial adenoviral systems encoding checkpoint-blocking modules along with additional immune-active payloads. In non-small cell lung cancer models, MSCs carrying a binary adenoviral platform composed of an oncolytic adenovirus and a helper-dependent adenoviral vector expressing IL-12, together with a PD-L1 blocker, enhanced HER2 CAR-T-cell infiltration, expansion, and effector function, with increased IFN-γ, granzyme B, and perforin production (208, 212). Mechanistically, this design indicates that the contribution of MSC-based checkpoint-blocking delivery may extend beyond local inhibition-receptor blockade alone and may be strengthened by the parallel integration of cytokine signaling or oncolysis on the same platform. However, at present, direct preclinical support remains concentrated in PD-1/PD-L1-focused systems, and a broader extension to other checkpoint pathways requires further validation across tumor types and delivery formats.

### 6.5 CAR-engineered MSCs as antigen-directed and functionally programmable platforms

With the continued development of MSC-based engineering strategies, the incorporation of chimeric antigen receptor (CAR) constructs has emerged as a potential approach to enhance the specificity and functional precision of MSC-mediated tumor targeting (213). In contrast to earlier designs that relied primarily on intrinsic tumor tropism or constitutive expression of therapeutic payloads, CAR engineering introduces antigen-directed recognition, potentially improving MSC localization and retention within tumor tissues (**Figure 4E**). Although this concept was inspired by the success of CAR T-cell therapy, its application in MSCs serves a distinct purpose. Rather than reproducing the direct cytotoxic activity of engineered lymphocytes, CARs in MSCs are generally used to refine the targeting capacity and facilitate the spatially restricted delivery of therapeutic factors within the TME.

Emerging preclinical evidence indicates that CAR incorporation can extend MSC functionality via several mechanisms. CAR expression enhances the interaction between engineered MSCs and antigen-positive tumor cells, thereby improving local retention and increasing the effective concentration of the delivered therapeutic agents. This feature is particularly relevant in models in which MSCs are engineered to express pro-apoptotic ligands, such as TRAIL, where CAR-mediated targeting improves tumor cell engagement and augments antitumor efficacy (213). In addition, limited evidence suggests that CAR signaling may influence MSC behavior beyond passive targeting, including modulation of their secretory or immunoregulatory properties in an antigen-dependent context; however, this aspect remains incompletely characterized and may vary depending on the CAR design and cellular context (214, 215). More recent approaches have explored the integration of CAR recognition with additional therapeutic modules, including immunoregulatory or drug delivery modules, supporting the feasibility of combining antigen specificity with multifunctional MSC engineering (216). Collectively, these results suggest that CAR-engineered MSCs may function as targeted delivery platforms with enhanced localization and improved functional control.

Despite these advances, several challenges limit the translational interpretation of CAR-MSC strategies. MSCs are inherently heterogeneous and exhibit context-dependent functional plasticity, which may influence therapeutic outcomes following genetic modification (1). Furthermore, the relationship between CAR engagement and downstream MSC responses remains insufficiently defined, and it is unclear whether CAR signaling can be consistently translated into predictable therapeutic effects *in vivo* (217). Additional considerations include limited persistence after administration, variability in manufacturing, quality control, and the need for robust safety mechanisms to prevent unintended effects. Accordingly, while current studies support the feasibility of CAR-based MSC engineering, the available evidence remains largely preclinical and insufficient to establish its broad clinical applicability. Future developments will likely focus on integrating antigen-directed targeting with more precisely regulated downstream functions, including strategies that enable MSCs to introduce defined immunological or antigenic signals within the TME, thereby providing a conceptual foundation for subsequent approaches based on tumor-associated antigen delivery and *in situ* immune priming.

### 6.6 Synthetic biology-enabled control circuits for programmable cargo release

In MSC engineering, the main objective of these strategies is not simply to add another cargo but to place cargo expression or release under conditional control so that therapeutic activity is preferentially initiated within the TME or in response to an external trigger (**Figure 4F**). Promoter performance depends on several design variables, including the choice of core promoter, HRE copy number, spacing, and degree of basal leakage. In parallel, inflammation-responsive switches and tumor protease-activated systems extend this logic to other tumor-associated inputs. Synthetic biology offers a framework for designing therapeutic cells with regulated rather than constitutive activities. By combining modular genetic elements, defined input–output relationships, and engineered control circuits, it is possible to improve the specificity, controllability, and therapeutic index of cell-based cancer therapy (218, 219). This concept is particularly relevant for MSC-based delivery, in which tumor tropism provides spatial targeting, whereas synthetic control systems may further restrict the timing and strength of payload production (220).

Among the endogenous regulatory strategies, hypoxia-responsive transcription remains one of the most directly relevant designs for tumor-targeted gene control (221). Hypoxia-inducible systems are typically constructed by coupling hypoxia-responsive elements (HREs) derived from HIF-responsive genes to a basal promoter, thereby restricting transgene expression to HIF-active regions. Earlier studies have shown that such systems can be used to control suicide genes, including HSV-TK and cytosine deaminase, in gene-directed enzyme–prodrug therapy, and can also be combined with radiotherapy-responsive elements to increase tumor selectivity. To improve specificity, dual-control systems combining HREs with tissue- or tumor-selective promoters and post-transcriptional or post-translational regulatory layers have been proposed (222). Recently, a hypoxia response promoter was engineered directly in human umbilical cord-derived MSCs, indicating that this type of regulation can be adapted to MSC-based therapeutic platforms (10). NF-κB-responsive circuits provide an example of inflammation-coupled transcriptional control, whereas protease-cleavable masking strategies, exemplified by tumor-activated IL-12 formats, illustrate how highly potent immune cargoes may remain functionally silent in the periphery and become activated only in protease-rich tumor tissue (223). Optogenetic designer cell systems have demonstrated that light-inducible circuits can control the local release of immunoregulatory molecules with high temporal resolution and antitumor activity *in vivo*. Sonogenetic platforms provide a related strategy using ultrasound as a noninvasive input to regulate engineered therapeutic cells (224, 225). At present, these externally actuated systems have been demonstrated more clearly on broader synthetic biology platforms, including engineered immune cells and bacteria, than in MSC-based tumor therapy. Nevertheless, they are relevant to MSC engineering because they define a plausible route for combining tumor tropism with an externally programmable output.

Externally controlled systems add another level of regulation by enabling on-demand payload activation. Overall, the significance of this area lies in shifting MSC-based delivery from passive cargo transport toward conditionally regulated therapeutic release to reduce constitutive off-tumor activity and improve the precision of local intervention.

## 7 Clinical perspectives and challenges

An analysis of records from ClinicalTrials.gov revealed that 27 clinical trials evaluating MSC-based therapies for cancer have been registered worldwide. Among these, 10 trials have been completed, with most studies being early-phase, classified as Phase I or Phase I/II (**Table 2**). The registered trials cover a broad spectrum of tumors, including glioblastoma, head and neck cancers, pancreatic cancer, prostate cancer, ovarian cancer, colorectal cancer, melanoma, lymphoma, and hematologic malignancies. Notably, many studies focusing on solid tumors have utilized MSCs as delivery vehicles for inflammatory cytokines, suicide genes, oncolytic viruses, and similar therapeutic agents. In contrast, for hematologic malignancies, the primary approach has been to leverage the immunomodulatory functions of MSCs to manage graft-versus-host disease following hematopoietic stem cell transplantation. Despite this diversity, the overall progress remains in the early-to-intermediate stages of clinical translation. The following section discusses the limited advancement of MSC-based cancer therapies in detail.

**Table 2:** Clinical trials of MSC-based cancer therapy.

NCT number	Study status	Types of tumors	Phases	Source of MSCs	Combined treatment
NCT02530047	Completed	Ovarian Cancer	1	Bone marrow derived MSCs	INF-β
NCT06446050	Recruiting	Advanced Colorectal Cancer	Early 1	Umbilical cord derived MSCs	CXCL chemokine and TNF superfamily co-stimulatory molecule-modified
NCT02079324	Completed	Head and Neck Cancer	1	NA/	GX-051 (IL-12)
NCT07143812	Recruiting	Glioblastoma	1	Bone marrow MSCs	Suicide gene
NCT04657315	Completed	Recurrent Glioblastoma	1,2	N/A	suicide gene, cytosine deaminase
NCT02068794	Active no recruiting	Recurrent Ovarian, Primary Peritoneal or Fallopian Tube Cancer	1,2	Adipose derived MSCs	Oncolytic measles virus encoding thyroidal sodium iodide symporter
NCT04758533	Active no recruiting	Diffuse Intrinsic Pontine Glioma	1,2	Bone marrow MSCs	Oncolytic Adenovirus (ICOVIR-5)
NCT05047276	Unknown	Uveal Melanoma, Metastatic	1,2	Bone marrow MSCs	Oncolytic Adenovirus (ICOVIR-5)
NCT01844661	Completed	Metastatic and Refractory Tumors	1,2	Bone marrow MSCs	Oncolytic adenovirus (ICOVIR5)
NCT03896568	Recruiting	Recurrent High-Grade Glioma	1	Bone marrow MSCs	Oncolytic adenovirus (DNX-2401)
NCT02145923	Unknown	Lymphoma	1,2	Bone marrow MSCs	Chemotherapy
NCT02270307	N/A	Leukemia, multiple myeloma	2, 3	N/A	Cyclophosphamide
NCT07048314	Not yet recruiting	Prostate Cancer	1,2	Adipose derived MSCs	Radical retropubic prostatectomy
NCT03106662	Completed	Hematological Malignancies	3	Bone marrow derived MSCs	Allogeneic hematopoietic stem cell transplantation
NCT01092026	Completed	Hematological Malignancies	N/A	Cord blood derived MSCs	Allogeneic hematopoietic stem cell transplantation
NCT00504803	Completed	Hematological Malignancies	2	Cord blood derived MSCs	Allogeneic hematopoietic stem cell transplantation
NCT01045382	Terminated	Leukemia, Lymphoma	2	N/A	Allogeneic hematopoietic stem cells
NCT05672420	N/A	Hematologic Neoplasms	1, 2	Umbilical cord derived MSCs	Autologous hematopoietic stem cell transplantation, consolidation therapy, re-induction therapy
NCT00827398	Completed	Hematological Malignancies	1, 2	N/A	Allogeneic hematopoietic stem cells
NCT05855707	Not yet recruiting	Hematological Malignancies	1	Umbilical cord derived MSCs	Allogeneic hematopoietic stem cells
NCT00447460	N/A	Hematological Malignancies	1, 2	N/A	Allogeneic hematopoietic stem cells
NCT01754454	N/A	Hematological Malignancies	1, 2	Umbilical cord derived MSCs	Allogeneic hematopoietic stem cells
NCT03847844	Completed	Hematological Malignancies	1, 2	Umbilical cord derived MSCs	Allogeneic hematopoietic stem cells
NCT02557724	Completed	Liver Neoplasm	N/A	Peripheral blood-derived MSCs	Liver transplantation
NCT01983709	Terminated	Prostate Cancer	1	Bone marrow derived MSCs	N/A
NCT04087889	No longer available	Pancreatic Cancer	N/A	Adipose derived MSCs	N/A
NCT05789394	Recruiting	Recurrent Glioblastoma or Recurrent Astrocytoma	1	Adipose derived MSCs	N/A

*Data sources:* ClinicalTrials.gov.

### 7.1 Clinical applicability: addressing tumor delivery barriers

From a clinical perspective, the appeal of MSC-based strategies lies less in introducing an entirely new antitumor agent and more in providing a delivery framework for therapeutic modalities whose efficacy has long been constrained by limited tumor access and microenvironmental restrictions. Across solid tumors, abnormal vascular architecture, dense stromal barriers, elevated interstitial pressure, and immune exclusion frequently converge to create a translational bottleneck in which therapeutic agents fail to reach the relevant intratumoral compartments in sufficient amounts or cannot be retained long enough to exert durable biological effects (226, 227). This challenge becomes especially evident in brain tumors and brain metastases, where the blood-brain and blood-tumor barriers restrict exposure, as well as in peritoneal dissemination and immunologically cold tumors, where compartmentalized fluid environments, stromal shielding, and deficient immune activation further limit therapeutic responsiveness (228).

The tumor-tropic behavior of MSCs and MSC-derived EVs provides a mechanistic basis for localizing effector modules within difficult-to-treat lesions, as supported by preclinical studies including oncolytic virus delivery in ovarian cancer and EV-based KRAS targeting in pancreatic cancer (203, 229). To overcome delivery barriers, locoregional administration (intratumoral/intraperitoneal) and chemotaxis engineering (e.g., CXCR2/CXCR4 overexpression) are actively pursued, while MSC-derived EVs offer a cell-free alternative with deeper tissue penetration. Collectively, these results suggest that the clinical relevance of MSC platforms lies in converting spatially inaccessible tumors into intervention-responsive settings.

### 7.2 Long-term safety

Discussions on the potential risks of MSCs in patients with cancer frequently cite patients with GVHD as a clinical reference. Following allogeneic hematopoietic stem cell transplantation, these patients experience incomplete immune reconstitution, coexisting inflammation, immunosuppression, and elevated baseline risks of recurrence, secondary tumors, and infections (230, 231). Recent clinical meta-analyses and systematic reviews have supported the good short-term tolerability of MSCs in GVHD. However, the significant variability in response rates and survival benefits across studies suggests that factors, such as the source of MSCs, preparation process, timing of administration, disease stratification, and concomitant immunosuppressive regimens, significantly affect clinical reproducibility (232). Notably, regulatory milestones have been achieved, and the U.S. The FDA has approved a bone marrow-derived MSC product for steroid-refractory acute GVHD in children. This indicates that MSC therapy can enter standardized clinical pathways when clear indications, controllable processes, and robust evidence are available.

The persistent controversy over tumor risk stems from difficulty separating this risk from confounding factors such as baseline recurrence risk and transplant-related medications (232). Long-term risks require consistent outcome definitions, sufficient follow-up, and rigorous stratified analysis (233). To mitigate theoretical immune surveillance impairment, strict patient selection (low/minimal residual disease) and inducible safety switches are promising solutions. Additionally, short-acting platforms (MSC-derived EVs or mRNA-engineered MSCs) and standardized long-term follow-up protocols (≥5 years) will help definitively isolate MSC-attributable risks from confounding factors.

### 7.3 Preclinical to early clinical development of engineered MSCs

Engineered MSCs have shifted from “cell therapy” to “migratory local delivery platforms”, confining effector molecule release within tumors to preserve efficacy while reducing systemic toxicity. Current engineering approaches include expressing immunomodulatory factors (e.g., IL-12, type I IFN), pro-apoptotic ligands (e.g., TRAIL), and serving as carriers for oncolytic viruses (234). However, first-pass pulmonary retention and rapid clearance following intravenous infusion remain major bottlenecks, making locoregional delivery routes (intratumoral/peritumoral) preferable with intratumoral pharmacodynamic readouts as core evidence (166).

MSCs can partially shield viral particles from rapid peripheral clearance and transport them to tumor margins or perivascular niches (235). Subsequent local amplification relies on intratumoral viral replication and lysis. Crucially, viral infection and lysis often trigger the release of immunogenic cell death signals and enhanced antigen presentation, thereby promoting dendritic cell maturation, T cell recruitment, and restoration of effector function, creating a sequential effect of “direct oncolysis followed by immune remodeling.” In MSC-mediated oncolytic virus delivery, variables affecting efficacy include MSC source variability, loading methods, release kinetics, and host antiviral immunity (10).

Despite rapid preclinical accumulation, clinical advancement remains slow. To accelerate translation, standardized potency assays (quantitative, mechanism-relevant bioassays) are needed to eliminate donor variability. Additionally, combination with immune checkpoint inhibitors (ICIs) represents the most clinically mature path forward. Locally delivered antitumor components can convert immunologically “cold” tumors into “hot” ones, and the subsequent addition of ICIs helps prevent T-cell exhaustion, thereby establishing a durable, long-term antitumor immune response.

## 8 Conclusion and future perspectives

The present review supports the central conclusion that the role of MSCs in cancer is determined primarily by context rather than by fixed intrinsic properties. Within tumors, MSCs are not uniformly tumor-promoting or tumor-suppressive but are highly plastic stromal components whose behavior is continuously shaped by chemokine gradients, inflammatory mediators, hypoxia-driven programs, and cell–matrix interactions in the TME. This context dependence helps explain the marked variability in homing efficiency, intratumoral persistence, and functional outcomes reported across studies and clarifies why native MSCs may contribute to immune suppression, angiogenesis, extracellular matrix remodeling, and therapeutic resistance under certain conditions, while exhibiting tumor-inhibitory effects only within narrower and less reproducible biological windows. From this perspective, the significance of engineering lies in converting stromal plasticity into defined and controllable therapeutic function. Through genetic modifications, synthetic payload installation, and microenvironment-responsive regulation, MSCs can be repositioned from biologically ambiguous stromal cells to programmable intratumoral delivery platforms capable of mediating localized cytotoxicity, immune activation, vascular interference, and TME remodeling. However, as the field advances, the principal barrier is increasingly shifting from mechanistic proof-of-concept to product feasibility. Therefore, progress will depend on aligning platform design with stratifiable indications, establishing verifiable evidence of biodistribution and effective intratumoral exposure, defining quantifiable pharmacodynamic endpoints, and developing manufacturing workflows that support batch consistency, release testing, and long-term safety evaluations.

Within this translational trajectory, two directions are particularly important. The first is the development of MSC-derived extracellular vesicles (MSC-EVs), particularly the small EV/exosome fraction, as controllable and formulable delivery systems. MSC-EVs offer a promising cell-free platform by preserving intercellular cargo transfer capacity while reducing the uncertainties associated with living cell therapies. However, their advancement requires tighter control over source-dependent heterogeneity, loading strategies, cargo composition, biodistribution, clearance, and batch consistency. The second is the more exploratory concept of using MSCs to install exogenous “Trojan” antigens within antigen-poor or highly heterogeneous solid tumors, thereby creating an actionable target for subsequent CAR-T or, more tentatively, TCR-T therapy. Although this concept is supported by the general logic of MSC-based tumor-directed delivery and independent studies showing that artificial target installation can sensitize tumors to engineered T-cell killing, its implementation within MSC platforms remains preliminary, and its extension to TCR-T therapy is even less mature because it requires efficient intracellular processing and peptide–MHC presentation *in situ*. Overall, future progress will likely depend on integrating programmable targeting, exposure-defined delivery, product-grade EV engineering, and rigorously quantifiable *in vivo* pharmacology into a unified translational framework. Thus, MSC-based and MSC-derived systems can be advanced from biologically variable entities to controllable, clinically developable therapeutic platforms.
